# Taxonomic guide and historical review of starfishes in northeastern Brazil (Echinodermata, Asteroidea)

**DOI:** 10.3897/zookeys.449.6813

**Published:** 2014-10-22

**Authors:** Anne Isabelley Gondim, Martin Lindsey Christoffersen, Thelma Lúcia Pereira Dias

**Affiliations:** 1 Universidade Federal da Paraíba, Programa de Pós-Graduação em Ciências Biológicas (Zoologia), Departamento de Sistemática e Ecologia, Laboratório de Invertebrados Marinhos Paulo Young, Bairro Cidade Universitária s/n, CEP. 58059-900, João Pessoa, PB, Brasil; 2 Universidade Estadual da Paraíba, CCBS, Departamento de Biologia, Laboratório de Biologia Marinha, Campus I, Rua Baraúnas, 351, Bairro Universitário, CEP 58429-500, Campina Grande, PB, Brasil

**Keywords:** Echinoderms, taxonomy, check list, sea-stars, northeastern Brazil

## Abstract

Presently more than 1900 species of sea stars are recognized, of which 77 are recorded for the coast of Brazil. Although the first starfish record in Brazil was published 363 years ago, our knowledge of this fauna remains unsatisfactory from a systematic and ecological point of view, particularly in the north and northeastern regions of the country. This study provides the first annotated list of sea stars from northeastern Brazil. Material described herein is housed at the collections of the Federal University of Paraíba, Federal University of Sergipe, and the Federal University of Bahia, Museum of Zoology of the University of São Paulo and Museu Nacional do Rio de Janeiro. Twenty-one species were identified, belonging to 12 genera, 10 families, and 5 orders. Descriptions of species are provided. Three new occurrences were recorded for northeast Brazil: *Astropecten alligator*, *Luidia ludwigi scotti*, and *Mithrodia clavigera*. Highest diversities of Asteroidea were encountered for the states of Bahia (n = 14 spp), Paraíba (n = 12 spp) and Pernambuco (n = 9 spp). No species were recorded for the states of Maranhão and Sergipe. Sandy substrates and depths below 10 m were the least sampled areas over the continental shelf. Herein we provide a first panorama on the fauna of Asteroidea occurring in the northeast region of Brazil, hopefully to function as a basic reference for biodiversity studies in this poorly studied area.

## Introduction

The Asteroidea represent the second most diverse group within the phylum Echinodermata, with an estimated number of 1900 living species (Mah and Blake 2012). Of these, 77 species are recorded for the Brazilian coastline (Ventura et al. 2012).

The first paper to deal with the Echinodermata of the Brazilian littoral was published by Georg Marcgraf (1648) and entitled “Natural History of Brazil”. In this work, animals observed from an expedition to northeast Brazil during the stay of the Count of Nassau are described, sometimes in a rather fanciful way (Hadel et al. 1999). Among the diverse groups described and illustrated by Marcgraf are two species of starfish (*Luidia senegalensis* as *Stella senegalensis*, and *Oreaster reticulatus*). Later C. F. Hartt collected 16 species of echinoderms from Abrolhos (Bahia), of which four were asteroids (*Oreaster reticulatus*, *Linckia guildingi*, Echinaster (Othilia) echinophorus, and *Coscinasterias tenuispina*) (Verrill 1868). Between 1875 and 1877 this same author collected another 62 species of echinoderms, which were described by Rathbun (1879). That paper furnished the first list of echinoderms from Brazil and in it 12 species of asteroids were recorded.

Thirty six years after the paper by Rathbun (1879), Verrill (1915) published a new list with descriptions and illustrations of 125 species of sea-stars from the West Indies, Florida, and Brazil. Later H. P. Oliveira (1940) furnished a new list of the Brazilian asteroids. Since then, several contributions on the marine benthic fauna were published by diverse authors, which increased our knowledge of the Brazilian asteroids. Concomitantly, several papers were published on the Echinodermata from Brazil by Dr. Luiz Roberto Tommasi. The paper published by Tommasi (1970) is his main contribution to the Class Asteroidea. In this work he produced descriptions and illustrations of the 42 species then known for the coast of Brazil.

After these pioneer studies, several works focusing on taxonomy, ecology and geographical distribution were developed, mainly in the south and southeast (e.g. Brito 1960, Carrera-Rodríguez and Tommasi 1977, Monteiro and Pardo 1991, Ventura and Fernandes 1995, Ventura et al. 1997, Nobre and Campos-Creasey 2000, Alves et al. 2002, Carvalho and Ventura 2002, Gibran 2002, Calil et al. 2009, Mariante et al. 2010).

For northeast Brazil, only three papers have focused on the Asteroidea: Lima-Verde and Matthews (1969), who studies the feeding habits of *Luidia senegalensis* in the State of Ceará; Manso (2006), who recorded the first Goniasteridae for the Potiguar Basin in the Cretaceous of Brazil, and Matthews and Lima-Verde (1969), who furnished ecological information on *Oreaster reticulatus* from the northeastern region. Knowledge on diversity along the northeastern littoral stems from species inventories. Among these: Lima-Verde (1969) recorded seven species along Ceará, Rio Grande do Norte, Pernambuco and Alagoas; Tommasi (1970) recorded six species for several northeastern states; Tommasi and Aron (1988) expanded to ten the known species from Bahia; Fernandes et al. (2002) record six species from Pernambuco; Magalhães et al. (2005) cited eight species for Bahia; Gondim et al. (2008) recorded five species for Paraíba; Gondim and Giacometti (2010) and Gondim et al. (2013) signaled two species for the coast of Piauí; and Miranda et al. (2012) recorded eight species from Alagoas.

Northeastern Brazil has a coastline with 3,400 km in extension (Pinheiro et al. 2008), which represents 42.5% of the entire Brazilian coastline. This region contains a great variety of ecosystems and has one of the largest reef environments of the Southwestern Atlantic Ocean. Notwithstanding, many areas remain unexplored (Marques and Lamas 2006) and our knowledge of the macrozoobenthos below 20 m is still limited, mainly regarding the outer platform and the continental slope (Migotto and Tiago 1999). Brazil has organized few marine expeditions (an example being the Programa de Avaliação do Potencial Sustentável de Recursos Vivos na Zona Econômica Exclusiva - REVIZEE), but most results continue concentrated in the south and southeastern regions, the addition of information for the north and northeastern regions being minimal below 20 m depth (Marques and Lamas 2006).

Although the first record of the Asteroidea for the Brazilian littoral was made about 363 years ago, the knowledge of this fauna in the north and northeastern regions still remains unsatisfactory and punctual, from both a systematic and an ecological point of view. The aim of the present work is to expand our knowledge on the morphology of the species of Asteroidea from northeastern Brazil.

## Materials and methods

Species determinations were based mainly on Tommasi (1970), Clark and Downey (1992), and Hendler et al. (1995). Synonyms were compiled from Tommasi (1970), Clark and Downey (1992), and Mah (2013). We provide a reference to the first work mentioning the synonym and to the main papers on the Brazilian fauna that subsequently cite the species.

Illustrations are based on photos made with a Canon A640 10MP camera coupled with a Nikon stereomicroscope. Morphometrics were recorded with a digital EDC 6 caliper ruler.

The studied material is conserved in 70% alcohol or preserved dry and registered in the visited collections.

### Study area

**Figure 1.**
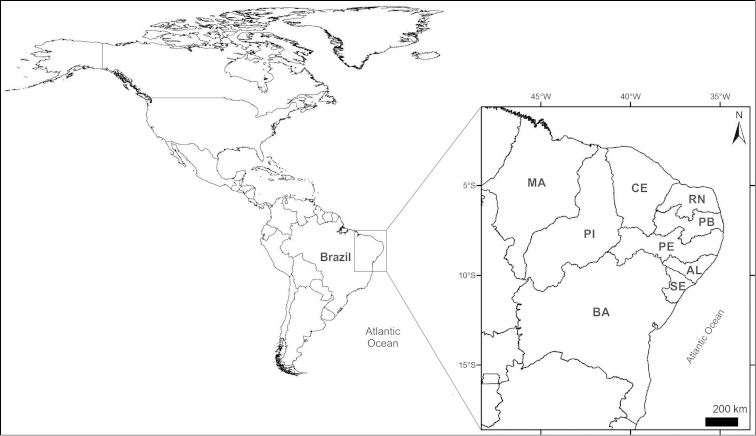
Map of the Americas showing the northeast region of Brazil divided in the nine states. **MA** Maranhão **PI** Piauí **CE** Ceará **RN** Rio Grande do Norte **PB** Paraíba **PE** Pernambuco **AL** Alagoas **SE** Sergipe, and **BA** Bahia.

The littoral region of northeast Brazil extends for approximately 3,400 km (Pinheiro et al. 2008), beginning in the Parnaíba River Delta and extending first to the east and then to the south up to the border of between the States of Bahia and Espírito Santo. The region encompasses nine coastal States: Maranhão, Piauí, Ceará, Rio Grande do Norte, Paraíba, Pernambuco, Alagoas, Sergipe, and Bahia (Fig. 1).

**Figure 2.**
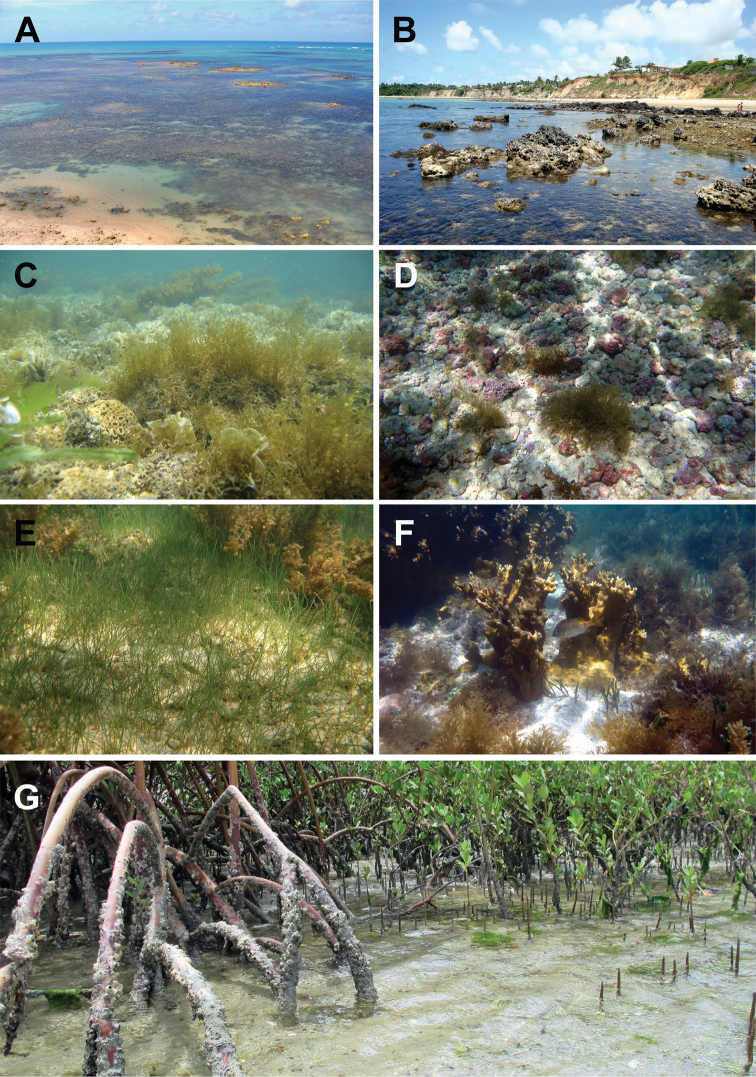
Some littoral environmnets and shallow-water biotopes used as habitats by sea stars in northeastern Brazil. **A** Fringing reef **B** Beach rocks **C** Algae banks **D** Rhodolith bed **E** Seagrass bed **F** Patch reef, and **G** Mangroves. Photos: Thelma LP Dias.

The region has a great diversity of littoral environments, such as beaches, dunes, cliffs, estuaries, deltas, sandbanks, reefs and underwater biotopes, such as rhodolith beds, seagrass beds and coral habitats (Fig. 2). They share Tertiary sediments from the Barreiras Formation, beach rocks, and coral reefs (Suguio 2003). This coastal diversity, allied to the favorable climatic conditions and year-round warm waters, makes this region one of the most important tourist destinations in the country (Pinheiro et al. 2008).

Due to the absence of large rivers and to the predominance of warm waters from the South Equatorial Current, offshore conditions in the northeast region are ideal for the formation of coastal coral reefs (Maury 2002). The most common formations along the coast are arenitic beach rocks (Mabesoone and Coutinho 1970), which harbour scleractinian corals and calcarious algae. Reefs composed exclusively of calcarious algae and corals also occur commonly. At the South of the State of Bahia, singular mushroom-shaped coral-reef formations, denominated locally as “chapeirões”, are present offshore (Abrolhos region).

The northeastern continental shelf is narrow and shallow. Due to the reduced continental influence and to the tropical climate, an important sedimentation of biogenic carbonates dominates most of the middle and outer platform, particularly between Macau (RN) and Maceió (AL) (Coutinho 2006). The platform ends very abruptly around depths of 60–80 m. The isobath of 20 m coincides mostly with the extent of the platform. The continental shelf may reach an extent of 60 miles at Cape São Roque (RN), and less than 5 miles in front of Recife (PE) (Kempf et al. 1970).

The biogenic carbonates form sand and gravel, consisting mainly of incrusting and ramified calcareous algae, with local occurrences of *Halimeda* spp. These sediments may contain up to 5% of carbonatic mud derived from the desintegration of larger organisms. In certain areas, these sediments undergo a process of litification, also affecting the quartsoze sands of the inner platform, originating the extensive coastal reefs known as beach rocks. These then become overgrown by algae and corals (Lana et al. 1996).

**Abbreviations:** Brazilian states – Ceará (CE), Piauí (PI), Rio Grande do Norte (RN), Paraíba (PB), Pernambuco (PE), Bahia (BA), Paraná (PR), Rio de Janeiro (RJ), Rio Grande do Sul (RS), Santa Catarina (SC), São Paulo (SP).

**Acronyms:** UFPB.Ech: Echinodermata Collection from Universidade Federal da Paraíba. MZUFBA: Museum of the Federal University of Bahia. MZUSP: Museum of Zoology of the University of São Paulo. MNRJ: National Museum of Rio de Janeiro.

## Results

A total of 21 species, belonging to five orders, 10 families and 12 genera were identified. These are listed and described below, following the taxonomic organization of Clark and Downey (1992).

### Checklist of Starfishes from northeastern Brazil

Phylum Echinodermata Brugière, 1791

Class ASTEROIDEA de Blainville, 1830

Order Paxillosida Perrier, 1884

Family Luidiidae Verrill, 1900

*Luidia alternata alternata* (Say, 1825)

*Luidia clathrata* (Say, 1825)

*Luidia ludwigi scotti* Bell, 1917

*Luidia senegalensis* (Lamarck, 1816)

Family Astropectinidae Gray, 1840

*Astropecten acutiradiatus* Tortonese, 1956

*Astropecten alligator* Perrier, 1881

*Astropecten brasiliensis* Müller & Troschel, 1842

*Astropecten cingulatus* Sladen, 1833

*Astropecten duplicatus* Gray, 1840

*Astropecten marginatus* Gray, 1840

Order Valvatida Perrier, 1884

Family Asterinidae Gray, 1840

*Asterinides folium* (Lütken, 1860)

Family Mithrodiidae Viguier, 1878

*Mithrodia clavigera* (Lamarck, 1816)

Family Oreasteridae Fisher, 1911

*Oreaster reticulatus* (Linnaeus, 1758)

Family Goniasteridae Forbes, 184

*Nymphaster arenatus* (Perrier, 1881)

*Plinthaster dentatus* (Perrier, 1884)

Order Velatida Perrier, 1884

Family Pterasteridae Perrier, 1875

*Calyptraster coa* Sladen, 1882

Family Ophidiasteridae Verrill, 1870

*Linckia guildingi* Gray, 1840

*Narcissia trigonaria* Sladen, 1889

Order Spinulosida Perrier, 1884

Family Echinasteridae Verrill, 1867

Echinaster (Othilia) brasiliensis Müller & Troschel, 1842

Echinaster (Othilia) echinophorus (Lamarck, 1816)

Order Forcipulatida Perrier, 1884

Family Asteriidae Gray, 1840

*Coscinasterias tenuispina* (Lamarck, 1816)

## Systematics

### Order Paxillosida Perrier, 1884

#### Family Luidiidae Sladen, 1889

##### Luidia alternata alternata

Taxon classificationAnimaliaPaxillosidaLuidiidae

(Say, 1825)

Figure 3a–d

Asterias alternata Say, 1825: 144–145.
Luidia alternata Lütken, 1859: 42–43. Brito 1968: 12–13, pl. 3, fig. 4. Tommasi 1970: 8, fig. 24. Tommasi and Aron 1987: 5. Tommasi et al. 1988: 6. Ventura et al. 2007: 236. Miranda et al. 2012: 9.
Luidia granulosa Perrier, 1869: 109–110, pl. 2, fig. 18.
Luidia variegata Perrier, 1875: 337.
Luidia numidica Koehler, 1911: 3, pl. 1, figs 8–11.
Luidia quequenensis Bernasconi, 1942: 253. Tommasi 1970: 8, fig. 23. Carrera-Rodriguez and Tommasi 1977: 62, 65.
Luidia bernasconiae A.H.Clark, 1945: 19–21.
Luidia alternata var. numidica Madsen, 1950: 206–209, fig. 9.
Luidia alternata numidica A.M.Clark, 1953: 388–389, pl. 41, fig. 1.
Luidia alternata alternata Clark & Downey, 1992: 8–9; Magalhães et al. 2005: 63.

###### Material examined.

Paraíba: 6°46'S; 34°50'W, 1spec., UFPB/ECH.877, 13.II.1981, 14m; 7°01'S; 34°41'W, 1spec., UFPB/ECH.876, 13.II.1981, 24m; 7°04"S; 34°41'W, 1spec., UFPB/ECH.879, 16.II.1981, 22m.

###### Type locality.

Dry Tortugas, Florida Keys, Florida (Clark and Downey 1992)–Neotype.

###### Description.

**Figure 3.**
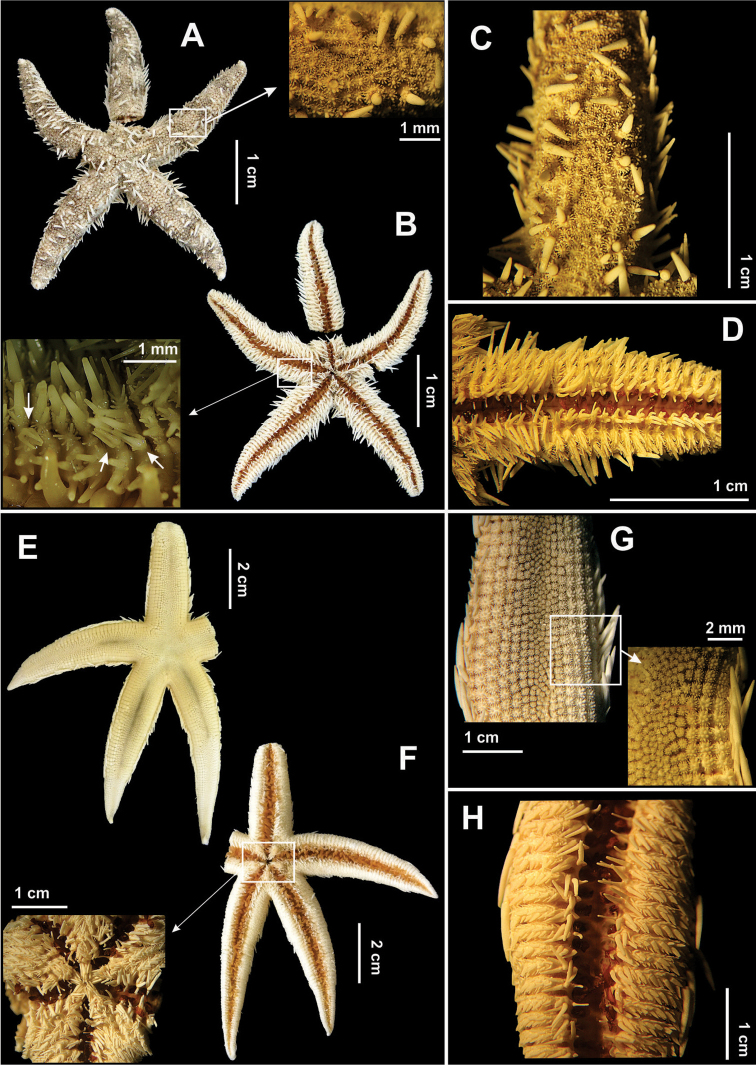
Some species of the family Luidiidae recorded in northeastern Brazil. *Luidia alternata alternata* **(A–D)**. **A** Abactinal view, in detail the paxilla **B** Actinal view, in detail the pedicellariae **C** Abactinal view of the arm **D** Actinal view of the arm; *Luidia clathrata* **(E–H)** **E** Abactinal view **F** Actinal view, in detail the mouth **G** Abactinal view of the arm, in detail the paxilla, and **H** Actinal view of the arm.

Body pentagonal (Fig. 3a, b). Five elongate and thin arms. Abactinal surface with paxillae (Fig. 3a). Carinal paxillae smaller than adradial paxillae, with 1–4 blunt central spinelets and with one or two series of marginal spinelets, numbering 12–20 per series. One of the central spines is slightly longer than remaining spines. Paxillae rounded and arranged into regular transversal rows. Some adradial paxillae with one long, conical and pointed central spine (~1.90 mm) (Fig. 3c). Inferomarginal fig with 1 or 2 long, thin spines (~1.76 mm), placed vertically and forming a well defined marginal row. Actinal surface with inferomarginal figs densely covered with spines of diverse shapes and sizes, there being 1–3 longer spines located centrally (Fig. 3c, d). Adambulacral fig with 4 spines placed vertically. The adambulacral spine is the smallest, being slightly curved and compressed. The two subambulacral spines are subequal in shape and size. A short spine is located laterally to the most external spine. Oral spines narrow and elongate. With bi, tri and tetravalvular pedicellariae on actinal surface (Fig. 3b).

**Colour.** Abactinal surface white or cream-coloured, with dark transversal bands. The colour of these bands varies, and may be brown, black, green, or purple. Actinal surface whitish or beige.

###### Distribution.

North Carolina, the Bahamas, Florida, Gulf of Mexico, Cuba, Jamaica, Honduras, Puerto Rico, Panama, Colombia, Venezuela, Brazil, Uruguay, and Argentina (Bernasconi 1943, Tommasi 1958, Downey 1973, Walenkamp 1976, Clark and Downey 1992, Hendler et al. 1995, Ventura et al. 2007, Alvarado et al. 2008). In Brazil from AL, BA, RJ, and SP (Verrill 1915, Brito 1962, 1968, Tommasi 1970, Magalhães et al. 2005, Miranda et al. 2012). This is the first record of the species for the coast of Paraíba. From 1 to 200 m in depth (Clark and Downey 2002), most common between 10 and 30 m.

###### Remarks.

The main characters distinguishing *Luidia alternata alternata* from the remaining species of the genus that occur in the Western Atlantic are their colour and the presence of long conical spines on the adradial paxillae. The abactinal colouration is distinctive. The nominal subspecies differs from its congeneric form *Luidia alternata numidica* Koehler, 1911, from West Africa, for attaining a larger size and for having longer paxillar spines. Clark (1982) provided a good discussion on the synonymy of the nominal subspecies and designates a neotype. Specimens analysed in this study are young individuals that may attain 200 mm along its larger ray. Furthermore, our specimens presented no morphological variations, agreeing with the descriptions of this species. Despite not receiving much attention in recent taxonomic surveys, pedicellariae are an important taxonomic character to distinguish species of asteroids. Clark (1982) and Clark and Downey (1992) furnished excellent illustrations of the species of *Luidia* known from the Atlantic.

###### Ecological notes.

This subspecies is primarily associated with sandy and muddy bottoms. It may also be found in mangroves or associated with fragments of shells and calcareous algae (Clark and Downey 1992, Benavides-Serrato et al. 2011). According to Hendler et al. (1995), the subspecies does not occur in large numbers, but is often found among the accompanying fauna in trawling nets. *Luidia alternata alterna* is carnivorous, feeding on epifaunal organisms, especially other echinoderms.

##### Luidia clathrata

Taxon classificationAnimaliaPaxillosidaLuidiidae

(Say, 1825)

Figure 3e–h

Asterias clathrata Say, 1825: 142.
Luidia clathratta Lütken, 1859: 37–39. Rathbun 1879: 150. Bernasconi 1943: 6–7. Tommasi 1970: 8. Magalhães et al. 2005: 63.
Luidia clathrata Lütken, 1859: 37. Tommasi 1958: 9, pl. 2, fig. 1; 1970: 8, fig. 22. Brito 1962: 4; 1968: 11–12, pl. 2, fig. 2. Carrera-Rodriguez and Tommasi 1977: 63–64. Tommasi and Aron 1987: 3. Tommasi et al. 1988: 6. Magalhães et al. 2005: 63. Ventura et al. 2007: 237. Manso et al. 2008: 185, fig. 7a–e. Lima and Fernandes 2009: 58. Xavier 2010: 75.

###### Material examined.

Paraíba: 6°57'S; 34°41'W, 2 spec., UFPB/ECH.875, 12.II.1981, 26m.

###### Type locality.

Probably no longer existant (Clark and Downey 1992).

###### Description.

Five long and narrow arms (Fig. 3e, f). Abactinal surface paxillar. Lateral paxillae quadrangular, forming three regular rows (Fig. 3g). Carinal paxillae small, smaller than lateral paxillae. Dorsal paxillae rounded, small, with 1–6 short, blunt, central spinelets and 6–18 slender, marginal spinelets. Inferomarginal figs with two long, pointed, conical spines (~2.58 mm), positioned vertically, the inferior one the largest. Actinal surface with inferomarginal figs densely covered with flattened, lanceolate spines (Fig. 3f). A row of short, actinolateral figs, with 1–3 short, lanceolate, divergent spines. Ambulacral figs with three spines placed vertically (Fig. 3h). The adambulacral spine is the smallest, being slightly curved and compressed. Of the two subambulacral spines, the inner one is longer and thicker than the outer spine. Oral spines long (~1.32 mm), thin and pointed, forming dense tufts on the inner angle of the jaw (Fig. 3f). Ocular fig granulose and elongate.

**Colour.** Abactinal surface bluish-gray, frequently with a darker line occupying the carinal region of the arm and disk. Hendler et al. (1995) cite other colour patters for the abactinal surface: brown, rose and salmon. Actinal surface white or cream-coloured.

###### Distribution.

Bermuda, Gulf of Mexico, Belize, Honduras, Nicaragua, Panama, Colombia, Venezuela, and Brazil (Downey 1973, Clark and Downey 1992, Hendler et al. 1995, Alvarado et al. 2008, Benavides-Serrato et al. 2005). In Brazil: PE, BA, RJ, SP, and SC (Rathbun 1879, Bernasconi 1943, Tommasi 1958, 1970, Brito 1960, 1962, Walenkamp 1976, Magalhães et al. 2005, Lima and Fernandes 2009, Xavier 2010). This study records the species for the first time in the State of Paraíba. From intertidal to 175 m in depth (Ventura et al. 2007), being most common in depths under 40 m (Hendler et al. 1995).

###### Remarks.

*Luidia clathrata* differs from *Luidia alternata alternata* for not having the abactinal surface spinulose. It differs from *Luidia senegalensis* for having only 5 arms and from *Luidia ludwigi scotti* for not having pedicellariae. According to Walenkamp (1976), the number of central spinelets on the paxillae and of inferomarginal spines increases with ontological development, the maximum numbers being, respectively, 7 and 3. In this study the examined specimen had only two inferomarginal spines, and 1–6 central spinelets on the paxillae. These characters indicate a juvenile individual. According to Hendler et al. (1995), adult individuals may attain 20 to 30 cm in disk diameter. Knott and Hopkins (1998) recognized two morphotypes of *Luidia clathrata* for the Colombian Caribbean, one with a gray colour and the other with three colours, which were separated by Hopkins and Knott (2010) into *Luidia clathrata* and *Luidia lawrencei*. Those authors also established and described a neotype for *Luidia clathrata*.

###### Ecological notes.

This species lives in sandy or muddy areas with low hydrodynamism near the coast, such as bays and lagoons, and is also found in mangroves and regions with low salinity. Further away from the coast, it lives in substrates with sand, mud and gravel (Machado et al. 2008, Benavides-Serrato et al. 2011). It feeds on a great variety of prey, including molluscs, crustaceans, and ophiuroids (Hendler et al. 1995). Like *Luidia alternata* and *Luidia senegalensis*, *Ludia clathrata* is host for the small polychaete *Podarke obscura* Verrill, 1873, that lives in the interior of its ambulacral groove. *Luidia clathrata* may form dense populations, and is thus frequently captured in trawling nets used in shrimp fisheries (McClintock and Lawrence 1985, Hendler et al. 1995). Presently it is considered to be a species vulnerable to extinction along the Brazilian coast (Machado et al. 2008).

##### Luidia ludwigi scotti

Taxon classificationAnimaliaPaxillosidaLuidiidae

Bell, 1917

Figure 4a–d

Luidia scotti Bell, 1917: 8–9. Tommasi 1970: 8, fig. 25. Carrera-Rodriguez and Tommasi 1977: 62, 65–66.
Luidia doello-juradoi Bernasconi, 1941: 117; 1943: 8–11. Brito 1962: 3.
Luidia rosaurae Jonh & Clark, 1954: 142–145.
Luidia doello-juradol Brito, 1968: 12, pl. 3, fig. 5.
Luidia ludwigi Walenkamp, 1976: 32–37, fig. 9, pl. 2, figs 1–3, pl.4, fig. 3. Machado et al. 2008: 179–180. Xavier 2010: 75.
Luidia ludwigi scotti A.M.Clark, 1982: 171–173. Tommasi 1985: 3. Tommasi et al. 1988: 6. Manso 1989: 357.
Luidia rosaurae John & Clark, 1954: 142–145, pl. 6, fig. 1.

###### Material examined.

Paraíba: 6°39'05"S; 34°49'W, 1 spec., UFPB/ECH.878, 28.V.1981, 20m.

###### Type locality.

Rio de Janeiro, Brazil (Clark and Downey 1992).

###### Description.

**Figure 4.**
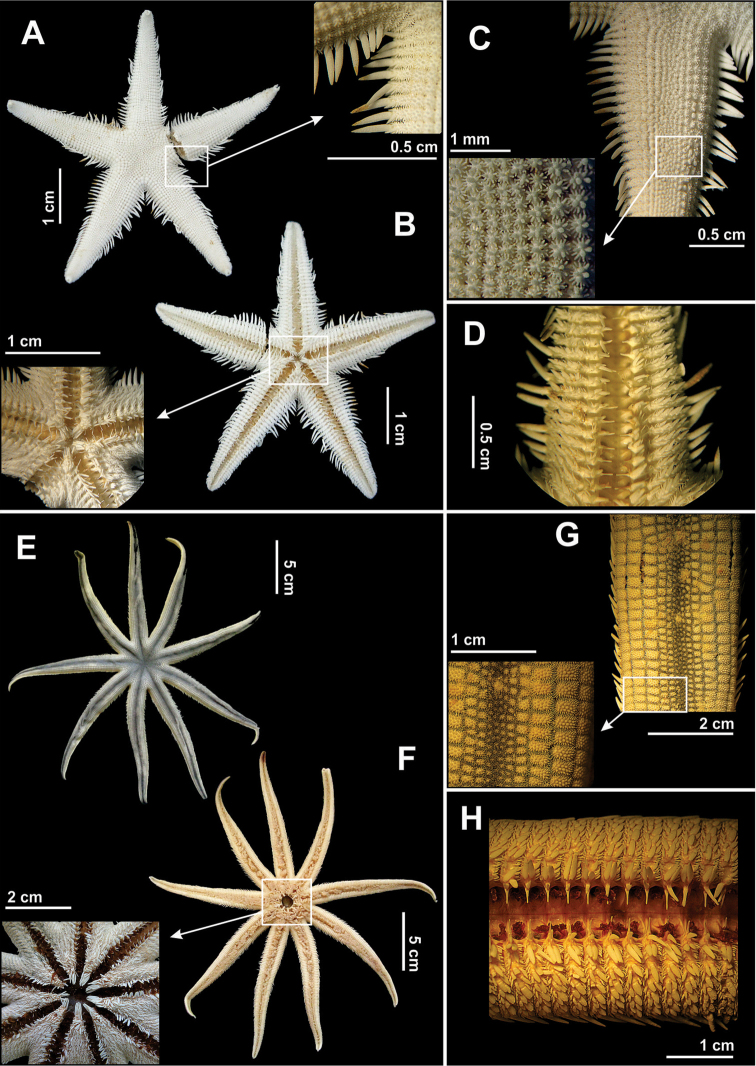
Some species of the family Luidiidae recorded in northeastern Brazil. *Luidia ludwigi scotti* **(A–D)** **A** Abactinal view, in detail abactinal intermediate area **B** Actinal view, in detail the mouth **C** Abactinal view of the arm, in detail the paxilla **D** Actinal view. *Luidia senegalensis* **(E–H)** **E** Abactinal view **F** Actinal view, in detail the mouth **G** Abactinal view of the arm, in detail the paxilla, and **H** Actinal view of the arm.

Body flattened. Five arms that taper gradually towards their extremities (Fig. 4a, b). Abactinal surface with paxillae (Fig. 4a). Dorsal paxillae small, ordered, with 1–6 central, short, blunt spinelets, and 18 marginal, slender, and denticulate spinelets (Fig. 4c). Adradial paxillae rectangular or quadrangular, slightly larger than the carinal paxillae. Inferomarginal figs with one pointed, elongate spine (~2.04 mm) forming the marginal fringe (Fig. 4a). The remaining part of the fig is covered by short, hyaline and denticulate spinelets. Actinal surface with inferomarginal figs, densely covered by flattened and lanceolate spines (Fig. b, d). Actinolateral fig with three short, slender, divergent spines. The median of these is the largest. The ambulacral spine long, flattened and slender (Fig. 4d). Bivalved pedicellariae present only on the actinal surface, mainly on the ventro-lateral figs and in the areas close to the arms, never occurring on the abactinal surface. Oral spines slender, long (~1.18 mm) with blunt tip, forming a bundle of spines at apex of jaw (Fig. 4c).

**Colour.** The colour pattern on the aboral surface of the body is very variable. Some specimens may be whitish with black spots on arms, others are brown with white spots on arms. Most, however, have the arms more or less banded with alternating white and brown stripes (Walenkamp 1976). Benavides-Serrato et al. (2011) and Clark and Downey (1992) observed specimens with dark pink spots in the center of the dorsal surface of the disc and transversal bands of the same tone on the arms. Oral surface with colour varying between white and cream. When observed in alcohol they are uniformly white.

###### Distribution.

Florida, Gulf of Mexico, Colombia, Venezuela, Guyana, French Guyana, Brazil, and Argentina (Mar del Plata) (Bernasconi 1943, Tommasi 1970, Clark and Downey 1992, Benavides-Serrato et al. 2005). In Brazil: RJ, SP, and SC (Brito 1962, 1968, Xavier 2010). This is the first record of the species for the littoral of northeast Brazil. From 20 to 126 m in depth. Clark and Downey (1992) considered the previous record at 5 m for the State of São Paulo to be doubtful. Thus the present study expands the bathymetric distribution to 20 m deep, previously established at 33–126 m.

###### Remarks.

This subspecies may be distinguished from other taxa in the genus by the presence of pedicellariae with three or four well-developed valves on the actinal surface, the delicate structure of the paxillae, and the slightly triangular shape of the arms, which become narrow distally. *Luidia clathrata* is similar to *Luidia ludwigi scotti*, but may be distinguished from it by having a stronger actinal skeleton and by the absence of pedicellariae. The synonymy between *Luidia rosaurae* John & Clark, 1954 and *Luidia scotti* was proposed by Clark (1982), who observed that the differences in the inferomarginal spines previously established between the two species were insignificant and unable to support the independence of the two species. Furthermore, with the observations of Walenkamp (1976) that *Luidia rosaurae* is conspecific with *Luidia ludwigi* Fisher, 1906, Clark and Downey (1992) recognized the subspecies *Luidia ludwigi scotti* for specimens from the Atlantic, as these have a paxillar arrangement that is distinct from the remaining species previously cited. According to Clark and Downey (1992), the relationships between *Luidia patriae* Bernasconi, 1941 and this subspecies still need to be investigated. The specimen analised in this study, even though representing a juvenile individual, did not present significant morphological variations when compared with the characters described for adult specimens, indicating that the morphological characters of *Luidia ludwigi scotti* do not vary significantly during ontogeny.

###### Ecological notes.

This subspecies occurs in non-consolidated sediments containing fine or coarse sand (Machado et al. 2008). In contrast to other taxa of *Luidia*, which do not present prey selectivity, in *Luidia ludwigi scotti* only eight different food types have been recorded, of which bivalves, foraminiferans and ophiuroids are their main prey (Benavides-Serrato et al. 2011). The presence of different prey species of distinct sizes in the stomachs of *Luidia ludwigi scotti* probably reflects a response to competition and coexistence with other species of sea-stars (Brögger and Penchaszadeh 2008).

##### Luidia senegalensis

Taxon classificationAnimaliaPaxillosidaLuidiidae

(Lamarck, 1816)

Figures 4e–h , 12a

Asterias senegalensis Lamarck, 1816: 567.
Luidia senegalensis Müller & Troschel, 1842: 78. Tommasi 1958: 9, pl. 2, fig. 1; 1970: 8, fig. 21; 1985: 3. Brito 1968: 10–11, pl. 3, fig. 1. Lima-Verde 1969: 10. Nomura and Fausto Filho 1966: 19. Fernandes et al. 2002: 422. Magalhães et al. 2005: 63. Manso et al. 2008: 185, fig 8c–e. Lima and Fernandes 2009: 58. Xavier 2010: 75.
Luidia marcgravii Steenstrup in Lütken 1859: 43–46.

###### Material examined.

Rio Grande do Norte: Timbau Beach, 3 spec., UFPB/ECH.1582, 28.II.1980; Areia Branca, Ponta do Mel, 1spec., UFPB/ECH.1428, 23.VI.1982. Paraíba: Lucena, Costinha Beach, 5 spec., UFPB/ECH.1673, 08.XI.2003; Cabedelo, Miramar Beach, 4spec., UFPB/ECH.1256, 03.II.1983; Cabedelo, Santa Catarina Beach, 1spec., UFPB/ECH.1583, 18.V.2007; Paraíba do Norte River Estuary, 1spec., UFPB/ECH.89, 18.06.1980; 1spec., UFPB/ECH.1586. 13.VI.1983.

###### Type locality.

Supposedly Senegal (‘L’ océan d’Afrique, les côtes du Senegal’), but probably West Indies (Clark and Downey 1992).

###### Description.

Body flattened. Disk rounded. Nine long and narrow arms (rarely 7) (Fig. 4e, f). Paxillae on abactinal surface. Paxillae small, arranged irregularly, occupying center of disk and of arms. Paxillae of carinal regions rounded, with 1–4 central, short, rounded spinelets and 12–16 marginal spinelets disposed into two rows. Adradial paxillae quadrangular, disposed in regular longitudinal and tranversal rows, bearing 4–10 central, short, rounded spines (Fig. 4g). Inferomarginal figs with two short, conical and slightly compressed spines (~2.20 mm), the upper one being the smallest. Towards the mouth the inferomarginal figs are covered by short, lanceolate spines (Fig. 4f). Between these and the lateral margins slender spinelets occur. Adambulacral figs with four spines, two elongate, compressed and slightly curved adambulacral spines, and two elongate, flattened and lanceolate subambulacral spines (Fig. 4h). Oral spines long (~2.59 mm) and thin, forming a dense tuft of spines on the apex of jaw. Ocular figs well developed and grunuliform. Pedicellariae absent.

**Colour.** Dorsally bluish-grey or greenish-gray, with a strong dark line along the central region of the disk and of the arms. The actinal surface is white to cream-coloured.

###### Distribution.

Florida, Cuba, Jamaica, Honduras, Nicaragua, Costa Rica, Panama, Venezuela and Brazil (Tommasi 1958, Clark 1982, Abreu-Pérez et al. 2005, Alvarado et al. 2008, del Valle García et al. 2008). In Brazil: PI, CE, PB, PE, AL, BA, RJ, SP, and SC (Rathbun 1879, Verrill 1915, Bernasconi 1943, Krau 1950, Tommasi 1958, 1970, Brito 1962, 1968, Lima-Verde 1969, Fernandes et al. 2002, Magalhães et al. 2005, Gondim et al. 2008, 2013, Gondim and Giacometti 2010, Xavier 2010, Miranda et al. 2012). This study provides the first record for the coast of Rio Grande do Norte. From 1 to 64 m in depth, being rare below 40 m (Clark and Downey 1992).

###### Remarks.

Distinguished from the remaining species from Brazil by the presence of 7 to 9 arms. *Luidia barbadensis* Perrier, 1881, recorded for Bahamas, Florida, Gulf of Mexico and south Brazil is the closest species, but it may be distinguished from *Luidia senegalensis* for having 6 arms, inframarginal figs with two long and narrow spines, and the usual presence of pedicellariae. Walenkamp (1979) identified one specimen with 6 arms from Guyana as *Luidia senegalensis*. His identification was questioned for some time, but the presence of dark bands on the carinal region of the arms and disk and the depth of collection (32 m, while the minimum known depth of *Luidia barbadensis* is 73 m) confirm the validity of this identification (Clark and Downey 1992). Our observation of both juvenile (dd ≤ 15 cm) and adult individuals permitted the conclusion that morphological characters do not vary during ontogeny. Thus forms with less than 7 arms or more than 9 arms (this later condition never having been observed in nature) must be the result of failures during metamorphosis (Hotchkiss 2000). As for the remaining Paxillosida, the structure of the paxillae represent a key taxonomic character for the identification of species.

###### Ecological notes.

The species lives in environments of low hydrodynamism, in sediment containing sand, mud, or a combination of both of these (Hendler et al. 1995). It feeds mainly on molluscs, but also of other echinoderms such as sea-stars of the genus *Astropecten* and irregular echinoids, as well as ophiuroids, copepods, decapods, scaphopods, and polychaetes (Penchaszadeh and Lera 1983). Sometimes a small commensal crustacean (*Minyoceras angustus* Dana, 1852) is found in the interior of its ambulacrum. This crustacean was also observed in *Luidia clathrata* (Brito 1960). According to Hendler et al. (1995), individulas may attain 30–40 cm in disk diameter (dd), and attain sexual maturity when at 15 cm in diameter. Presently it is considered vulnerable to extinction along the Brazilian littoral. The main causes of population decline are its high succeptibility to the effects of pollution of the water column and the frequent accidental capture in trawling nets (Machado et al. 2008).

#### Family Astropectinidae Gray, 1840

##### Astropecten alligator

Taxon classificationAnimaliaPaxillosidaAstropectinidae

Perrier, 1881

Figure 5a–d

Astropecten alligator Perrier, 1881: 30.
Astropecten nuttingi Verrill, 1915: 181, pl. 12, fig. 2e, pl. 21, figs 1–2.

###### Material examined.

Paraíba: 7°01'S; 34°41'05"W, 1 spec., UFPB/ECH.881, 13.II.1981, 24m.

###### Type locality.

Alligator Reef, Florida Keys, Florida (Clark and Downey 1992).

###### Description.

**Figure 5.**
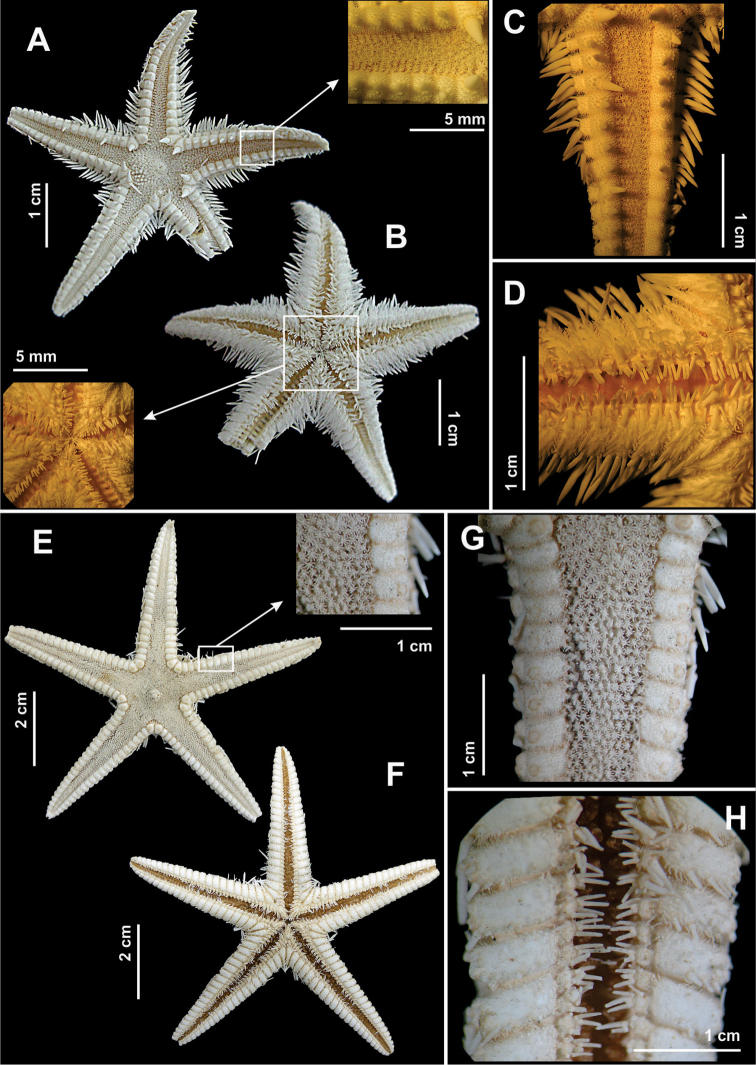
Some species of the family Astropectinidae recorded in northeastern Brazil. *Astropecten alligator* **(A–D)** **A** Abactinal view, in detail of the paxilla **B** Actinal view, in detail of mouth **C** Abactinal view of the arm **D** Actinal view of the arm; *Astropecten cingulatus* **(E–H)** **E** Abactinal view, in detail the paxilla **F** Actinal view **G** Abactinal view of the arm, and **H** Actinal view of the arm.

Body flattened dorso-ventrally. Disk small, with five long and narrow arms (~4.93 mm) (Fig. 5a, b). Epiproctal cone pronounced (this region of the disk and the surrounding areas are inflated) (Fig. 5a). Abactinal surface flat and covered by small paxillae disposed in regular rows (Fig. 5a). Paxillae with a central spinelet and 8–10 (usually 9) thick, marginal spinelets with blunts extremities. Superomarginal figs short (~1.14 mm) and narrow (~1.59 mm), covered by short and blunt spinelets, giving a granulose appearance to animals. Each of these figs has an elongate, conical and pointed spine (Fig. 5a, c), which has the same length from the interbrachial area to the extremity of each arm. Inferomarginal figs with two long, flattened and pointed spines, placed in parallel to each other. These figs are also covered by short and narrow spinelets. Towards the mouth the inferomarginal figs are covered by many flattened and pointed spines, which become elongated at the margins. Adambulacral figs with three long, flattened adambulacral spines, placed in parallel to each other, the median one being slightly longer than the other two (Fig. 5d). There are three to four long and flattened subambulacral spines, the median of which is longer and wider than the other two.

**Colour.** Reddish-brown dorsally, with conspicuous dark bands along the center of each arm (Clark and Downey 1992), or uniformly orange-red.

###### Distribution.

North Carolina, the Bahamas, Florida, Honduras, Nicaragua Colombia, and Brazil (Verrill 1915, Gray et al. 1968, Clark and Downey 1992, Benavides-Serrato et al. 2005, 2011, Alvarado et al. 2008). In Brazil: Benavides-Serrato et al. (2005) cite its occurrence in the north of Brazil. This study expands the distribution southward to the State of Paraíba, being the first reference for the northeast region of Brazil. From 22 to 576 m in depth (Benavides-Serrato et al. 2011), most frequent between 22 and 114 m (Clark and Downey 1992).

###### Remarks.

*Astropecten alligator* difers from the remaining species in the genus recorded for Brazil for presenting a single, elongate, erect conical spine on all the superomarginal figs. This character is present in both juvenile and adult individuals (dd = 41.0 mm). For a long time it was believed that Perrier (1881) had described *Astropecten alligator* based on a juvenile specimen and the recognition of this species remained uncertain. Clark and Downey (1992) analised the type material of *Astropecten alligator*. They were able to validate the species and consider *Astropecten nuttingi* Verrill, 1915 a junior synonym of this taxon. In a molecular phylogenetic analysis of *Astropecten*, Zulliger and Lessios (2010) concluded that *Astropecten alligator* and *Astropecten americanus* probably belong to the same species and that the species needs to be extensively revised.

###### Ecological notes.

Usually inhabits muddy environments, but may also be found associated to communities of azooxanthelate corals (Benavides-Serrato et al. 2011). It is common along the coast of Florida (Clark and Downey 1992), but for a long time it has been erroneously cited there as *Astropecten nuttingi*. In other areas *Astropecten alligator* is a rare species, being little cited in the literature.

##### Astropecten articulatus

Taxon classificationAnimaliaPaxillosidaAstropectinidae

(Say, 1825)

Asterias articulata Say, 1825: 144.
Astropecten dubius Gray, 1840: 182.
Asterias aranciaca Gould, 1841: 349 (non *Asterias aranciaca* Linnaeus, 1758).
Astropecten articulates Müller & Troschel, 1842: 72. Tommasi 1970: 6, fig. 17. Tommasi et al. 1988: 5. Manso 1989: 357.
Astropecten buschi Müller & Troschel, 1843 (a variety of *Astropecten articulatus* (Say, 1825) according to Doderlein (1917)).
Astropecten articulatus dubius Verrill, 1915: 165.
Astropecten articulatus var. valenciennesi A.H.Clark, 1939: 442.
Astropecten articulatus duplicatus Zoppi de Roa, 1967: 277, fig. 6. (non *Astropecten duplicatus* Gray, 1840).

###### Material examined.

Caraguatatuba, São Paulo, 1 spec., MZUSP484, 16.X.2001, 19m.

###### Type locality.

Florida (Clark and Downey 1992).

###### Description.

Body pentagonal, flattened dorso-ventrally. Five long and narrow arms (length of arm corresponds to approximately four times its width), which become gradually narrow towards tips. Abactinal surface with paxillae. Paxillae disposed regularly, carenals smaller than adradials. 1–6 short, thick and blunt paxillar spines in center, 10–16 in margins. Supermarginal figs granulose, with a large spine (~1.54 mm) positioned internally on the interbrachial figs. Some distal supermarginal figs have a short spine positioned more externally. Inferomarginal figs with two elongate, flattened and pointed marginal spines, positioned horizontally. Small oral spine. Three adambulacral spines divergent, the median one being the largest.

**Colour.** Dorsally dark blue or purple in paxillar region. Superomarginal figs white or orange. Oral surface white of beige (Hendler et al. 1995; Benavides-Serrato et al. 2011).

###### Distribution.

North Carolina, Florida, the Bahamas, Mexico, Cuba, Puerto Rico, Dominican Republic, Panama, Colombia, Venezuela, Brazil, and Uruguay (Tommasi 1970, Clark and Downey 1992, Hendler et al. 1995, Alvarado 2011). In Brazil: PI, CE, RJ, SP, and RS (Lima-Verde 1969, Netto 2006). From 0 to 550 m in depth, being most common between 5 and 20 m (Clark and Downey 1992, Hendler et al. 1995).

###### Remarks.

*Astropecten articulatus* differs from the congeneric *Astropecten cingulatus* for having a spine or tubercule on the distal supermarginal figs, two flat spines on each inferomarginal fig and subambulacral spines larger that the adambulacral spines. The original description of *Astropecten articulatus* Say (1825) is excelent, but the emphasis given by this author on the presence of spines and tubercules resulted in some uncertainty regarding the validity of *Astropecten duplicatus* (Gray, 1840) (Clark and Downey 1992). According to Clark and Courtman-Stock (1976) and Clark and Downey (1992) the confidence in the presence of spines on the superomarginal figs as a specific criterium is doubtful. As in other species of the genus, *Astropecten articulatus* presents a series of morphological variations. Verrill (1915) provides a good discussion of these variations, and stresses the differences found between juvenile and adult individuals. We have only been able to examine a single juvenile specimen, which nevertheless presented all the diagnostic characters of the adults.

###### Ecological notes.

Inhabits sandy environments. According to Benavides-Serrato et al. (2011) and Hendler et al. (1995), this species is common offshore over continental shelf, being particularly abundant in North Carolina. *Astropecten articulatus* is a voracious and non-selective predator (Hendler et al. 1995). Wells et al. (1961) recorded 91 food items for 124 specimens collected in North Carolina, gastropods, bivalves, and scaphopods being the most importante items. Small crustaceans, juveniles of *Mellita* sp. and *Astropecten articulatus* itself were also recorded as food items for the species, although in a lower level of importance (Hendler et al. 1995).

##### Astropecten brasiliensis

Taxon classificationAnimaliaPaxillosidaAstropectinidae

Müller & Troschel, 1842

Astropecten brasiliensis Müller & Troschel, 1842: 68. Tommasi et al. 1988: 5. Manso 1989: 357. Fernandes et al. 2002: 422. Netto 2006: 25–26, pl. 2a, fig. 16a. Ventura et al. 2007: 230. Machado et al. 2008: 350. Lima and Fernandes 2009: 58. Xavier 2010: 75. Miranda et al. 2012: 143, 144.
Astropecten braziliensis Rathbun, 1879: 150. Tommasi 1970: 6.
Astropecten brasiliensis riensis Döderlein, 1917: 84.
Astropecten brasiliensis armatus Jonh, 1948: 503.
Astropecten armatus brasiliensis Tortonese, 1956: 329. Tommasi 1958: 12–13, pl. 2, fig. 3; 1970: 7, fig. 19. Brito 1962: 3; 1968: 7–8, pl. 4, fig. 3. Lima-Verde 1969: 10. Carrera-Rodrigues and Tommasi 1977: 81–83. Magalhães et al. 2005: 63.
Astropecten brasiliensis brasiliensis Döderlein, 1917: 83.
Astropecten armatus riensis Tommasi, 1958: 13–14, pr. 2, fig. 4. Brito 1962: 3; 1968: 8, pl. 4, fig. 2.
Astropecten riensis Tommasi, 1970: 7. Carrera-Rodrigues and Tommasi 1977: 89. Tommasi and Aron 1988: 3. Tommasi et al. 1988: 5.

###### Material examined.

Ceará: Fortaleza, Mucuripe, 1 spec., MNRJ285, 1945. Rio Grande do Norte: Areia Branca, Ponta do Mel, 1 spec., UFPB/ECH.1919, 23.VI.182. Bahia: Salvador, 2 spec., UFBA00132, 01.III.2000.

###### Type locality.

São Sebastião Island, São Paulo, Brazil (Clark and Downey 1992).

###### Description.

Disk small with long, slender, dorsally flattened arms (~12.44 mm). Abactinal surface densely covered by overlapping and irregularly arranged paxillae. Paxillae with 26–30 long, blunt spinelets, the central ones sometimes shorter and more rounded than the marginal ones. Carinal paxillae larger than the adradial ones. Superomarginal figs longer (~5.7 mm) than wide (~1.19 mm), covered by short, apically rounded spinelets (giving them a granulose aspect), and having two large spines (rarely one). Inferomarginal figs with two long and flattened spines positioned vertically and forming a marginal fringe, the most ventral one being larger than the dorsal one. Adambulacral figs with three ambulacral spines, the inner one being slightly longer than the other two. Without pedicellariae.

**Colour.** According to Bernasconi (1957), live animals have an intense violet colour with the spines of the marginal fringe yellowish or pinkish. Actinal surface light salmon colour. When dry specimens may be light pink or whitish.

###### Distribution.

Honduras, Panama, Suriname, Brazil, Uruguay, and Argentina (Mar del Plata) (Tommasi 1958, 1970, Clark and Downey 1992, Alvarado et al. 2008, Ventura et al. 2007). In Brazil: CE, PE, AL, BA, RJ, SP, SC, and RS, incluing the islands of Fernando de Noronha and Trindade (Rathbun 1879, Verrill 1915, Tommasi 1958, 1970, Walenkamp 1976, Brito 1962, Lima-Verde 1969, Fernandes et al. 2002, Miranda et al. 2012). In this paper we establish the first record for Rio Grande do Norte. From 7 to 45 m in depth (Ventura et al. 2007).

###### Remarks.

This species differs from the remaining species of the genus known from the Brazilian coast for presenting paxillary spinelets and spines of the marginal fringe long and thin and for having up to two spines on the supermarginal figs. The vast synonymy presented by *Astropecten brasiliensis* evidences the plasticity of some of its characters, such as the number of spines on the superomarginal figs. This character, together with the shape of the marginal spines and paxillae spinelets formed the basis for the establishment of the five known subspecies. Döderlein (1917) considered *Astropecten armatus* Gray, 1840 and *Astropecten erinaceus* Gray, 1840 subspecies of *Astropecten brasiliensis* on the basis of differences in the adambulacral and superomarginal spines. Boone (1933), Clark (1940) and John (1948) disagreed with Döderlein (1917) and considered the three species not to be different, stating that the characters proposed to diagnose the species were not significant. Walenkamp (1976) listed a series of variations observed in specimens from Surinam and established *Astropecten brasiliensis* and *Astropecten riensis* as distinct species. Bernasconi (1957), Brito (1968) and Tommasi (1958, 1970) adopted the subspecies proposed by Müller and Troschel (1842) and Döderlein (1917) for Brazilian material. Clark and Downey (1992), analysing the neotype of the species, concluded that *Astropecten brasiliensis* and *Astropecten armatus* are distinct, while *Astropecten riensis* is a synonym of the first. Furthermore, he considered all the described subspecies to be synonyms. We agree with the proposal of Clark and Downey (1992) and include the subspecies (*Astropecten brasiliensis riensis*, *Astropecten brasiliensis armatus*, *Astropecten brasiliensis brasiliensis* and *Astropecten armatus riensis*) and *Astropecten riensis* as synonyms of *Astropecten brasiliensis*. The specimen examined in this study was broken, but its taxonomic characters were observable, except for the loss of the superomarginal spines. Scars of these spines remained on the figs, however, and we were able to establish that two spines occurred per fig, characterizing an adult individual.

###### Ecological notes.

As a rule burrowed in sand substrates, where it is a generalist predator, feeding on a variety of organisms from the benthic endofauna, such as bivalves, gastropods, crustaceans, echinoderms, and polychaetes (Ventura et al. 2007). As all species inhabiting soft sediments, *Astropecten brasiliensis* is suffering the impact of excessive collecting, being captured in bottom trawling nets and frequently do not resist the damage inflicted by these fishing efforts (Machado et al. 2008). Presently the species is considered to be vulnerable to extinction along the Brazilian coast.

##### Astropecten cingulatus

Taxon classificationAnimaliaPaxillosidaAstropectinidae

Sladen, 1833

Figure 5e–h

Astropecten cingulatus Sladen, 1883: 266. Brito 1962: 3; 1968: 9, pl. 4, fig. 4. Tommasi 1970: 5, fig. 16; 1985: 3. Carrera-Rodrigues and Tommasi 1977: 84–86. Tommasi and Aron 1987: 3. Manso 1989: 357. Tommasi et al. 1988: 5. Ventura et al. 2007: 231. Xavier 2010: 75.
Astropecten mesactus Studer, 1884: 46.
Astropecten jarli Madsen, 1950: 181.

###### Material examined.

Rio de Janeiro: Cabo Frio, 1 spec., MNRJ1853, 18.VI.1997.

###### Type locality.

Pernambuco, Brazil (Clark and Downey 1992).

###### Description.

Body pentagonal, flattened dorso-ventrally. Five long (~37.70 mm) and narrow (~9.56 mm) arms (length of arm corresponds to approximately four times its width) (Fig. 5e). Madreporite oval (~1.52 mm) and marginal. Epiproctal cone pronounced. Paxillae small and granulose, with 1–4 central spinelets and 9–12 marginal spinelets. Paxillar spinelets granulose (Fig. 5e). Superomarginal figs granulose (Fig. 5g). Two fringes of marginal spines aligned horizontally. First row of spines of inferomarginal figs with three marginal spines, disposed in parallel to each other, two being of the same length and one shorter. Second row with four elongate and slightly flattened spines, three of which are subequal in length and one much shorter. Oral spines short, forming a bundle at the apex of the jaw (Fig. 5f). Adambulacral figs with 3–4 divergent spines, the median one being the largest (Fig. 5h). Subambulacral spines forming a bundle of elongate and slightly flattened (lanceolate) spines. Pedicellariae rarely present.

**Colour.** According to Benavides-Serrato et al. (2011) and Bernasconi (1957), the species has the abactinal surface red or orange-red and the actinal surface white. Ventura et al. (2007) recorded a cream-colour on the dorsal surface and white on the oral surface in Brazilian specimens.

###### Distribution.

North Carolina, the Bahamas, Gulf of Mexico, Mexico, Nicaragua, Costa Rica, Panama, Colombia, Brazil, Uruguay, Argentina, and Africa (Tommasi 1970, Carrera-Rodríguez and Tommasi 1977, Clark and Downey 1992, Ventura et al. 2007, Alvarado et al. 2008). In Brazil: PE, RJ, SP, and SC, including the submarine banks and mountain ranges Vitória-Trindade and Vitória Island (SP) (Brito 1962, Tommasi 1970, 1985, Tommasi and Aron 1987, Manso 1989, Xavier 2010). Intertidal to 1350 m in depth (Clark and Downey 1992), being most frequent between 51 and 129 m (Carrera-Rodríguez and Tommasi 1977).

###### Remarks.

*Astropecten cingulatus* differs from its closest species, *Astropecten articulatus*, for having three rounded spines on each inferomarginal fig, and for having subambulacral spines that are smaller than the adambulacral spines and rounded. Bernasconi (1957) stresses a few morphological variations observed in specimens from Uruguay and Argentina. Among these are the aspect of the superomarginal figs, which do not have large spines, or then there are only a few small, granuliform spines on the first figs. According to Ventura et al. (2007) the specimens collected along the Brazilian coast have short arms, in contrast to the indication in the original diagnosis. The single individual we examined, on the other hand, corresponds to that indicated in the literature (lenght about four time its width).

###### Ecological notes.

This species lives on sandy or muddy bottoms of the littoral region up to depths of 50 m (Tommasi 1970, Machado et al. 2008). It feeds mainly on gastropods, bivalves, crustaceans, and cirripeds (Ventura et al. 2007). *Astropecten cingulatus*, and other species of the genus, are frequently captured in trawling nets and the species is presently included among those vulnerable to extinction in Brazil (Machado et al. 2008). According to Brito (1962), this species is abundant along the coast of Pernambuco, but no other works conducted in this state confirm this observation.

##### Astropecten marginatus

Taxon classificationAnimaliaPaxillosidaAstropectinidae

Gray, 1840

Figures 6a–e , 12b

Astropecten marginatus Gray, 1840: 181. Tommasi 1958: 14, pl. 2, fig. 5; 1970: 5, fig. 15. Brito 1962: 3; 1968: 7, pl. 4, fig. 1. Lima-Verde 1969: 11. Carrera-Rodrigues and Tommasi 1977: 88–89. Tommasi et al. 1988: 5. Nomura and Fausto Filho 1966: 19. Gondim et al. 2008: 155. Lima and Fernandes 2009: 58. Xavier 2010: 75.
Astropecten ciliatus Grube, 1857: 340.
Astropecten richardi Gary, 1840: 181.
Astropecten orans Sluiter, 1895: 54.

###### Material examined.

Rio Grande do Norte: Areia Branca, Ponta do Mel, 2 spec., UFPB/ECH.1842, 23.VI.1982. Paraíba: Cabedelo, Miramar Beach, 1 spec., UFPB/ECH.1840, 03.II.1983; João Pessoa, 7°7'23,3"S; 34°48'27,9"W, 1 spec., UFPB/ECH.1839, 14.IX.1980; João Pessoa, Tambaú Beach, 1spec., UFPB/ECH.864, 03.X.2007. Pernambuco: Goiana, Catuama, UFPB/ECH.1427, 1 spec., 31.X.1982.

###### Type locality.

Unknown (Clark and Downey 1992).

###### Description.

**Figure 6.**
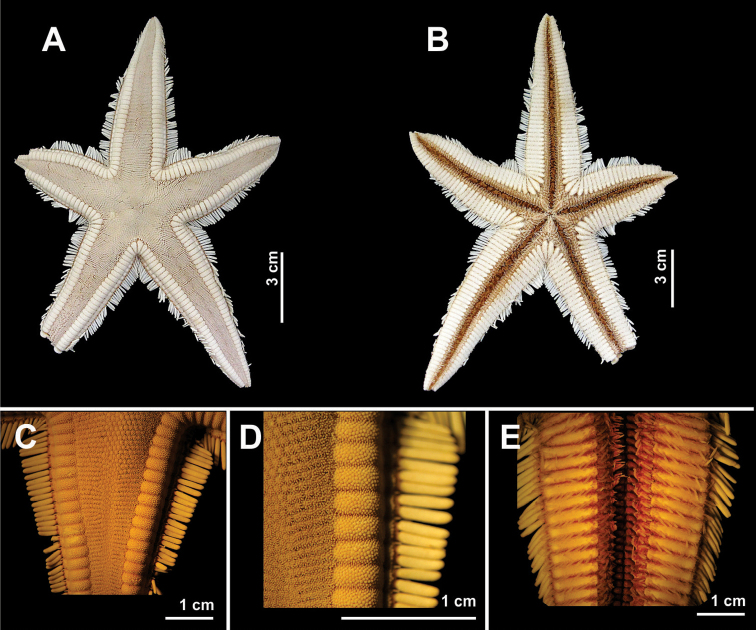
Some species of the family Astropectinidae recorded in northeastern Brazil. *Astropecten marginatus* **(A–E)** **A** Abactinal view **B** Actinal view **C** Abactinal view of the arm **D** Detail of the superomarginal fig, and **E** Actinal view of the arm.

Body flattened. Five broad, triangular arms (Fig. 6a, b). Abactinal surface covered by paxillae arranged in regular transversal rows. Circular madreporite positioned marginally in one of the abactinal intermediate areas. Carinal paxillae slightly smaller than the adradials (Fig. 6d). Paxillae small with 6–8 central spinelets and 12 marginal spinelets, all short and blunts. Superomarginal figs granulose, broader (~3.89 mm) than long (~2.00 mm) (Fig. 6c, d). Inferomarginal figs granulose, with two thick, blunt, parallel spines of similar length (~3.92 mm) and one small spine positioned laterally in relation to the other two. These spines form a well defined marginal bundle. Actinal surface with inferomarginal figs partially naked, having two series of marginal spines positioned laterally and one other marginal series with four thin, elongate, and flattened spines (~1.66 mm), located behind the aboral marginal row of spines (Fig. 6e). Adambulacral figs with a series of small spines on the proximal face and three long, narrow, and flattened adambulacral spines, the median of which is longest and widest. Six elongate, narrow, and pointed oral spines (~1.67 mm). Ocular fig small and bilobed.

**Colour.** Dorsally either blue with white margins (Clark and Downey 1992) or uniformly orange-coloured (Benavides-Serrato et al. 2011). Most specimens collected in northeastern Brazil vary from cream-coloured to grayish. Dried specimens become white.

###### Distribution.

Costa Rica, Puerto Rico, Colombia, Venezuela, Guyana, and Brazil (Tommasi 1958, Clark and Downey 1992, Hendler et al. 1995, Alvarado et al. 2008, Benavides-Serrato et al. 2005, 2011). In Brazil: CE, PB, PE, RJ, SP, SC, and RS (Bernasconi 1955, Brito 1962, Lima-Verde 1969, Netto 2006, Gondim et al. 2008, Lima and Fernandes 2009, Xavier 2010). This is the first record for the littoral of Rio Grande do Norte. From 1 to 130 m in depth (Clark and Downey 1992).

###### Remarks.

*Astropecten marginatus* differs from the remaining species of the genus known from Brazil for having large, triangular arms and two long, thick and blunt spines on each inferomarginal fig. Unlike other species of *Astropecten*, *Astropecten marginatus* shows little morphological variation in characters considered of taxonomic interest (Walenkamp 1976). We observed both juveniles (R = 11.73 mm) and adults (R = 81.41 mm).

###### Ecological notes.

This species lives in substrates containing sand or sand with mud (Ortega et al. 2010). As most species of the genus, it is an active predator with a generalist food diet (Ortega et al. 2010). This is one of the most common species in northeastern Brazil, being abundant below 2–4 m (Benavides-Serrato et al. 2011). Together with other species in the genus, populations of *Astropecten marginatus* suffer great collecting pressures from bottom trawling fishing nets. Presently it is included among the Brazilian species vulnerable to extinction (Machado et al. 2008).

### Order Valvatida Perrier, 1884

#### Family Asterinidae Gray, 1840

##### Asterinides folium

Taxon classificationAnimaliaValvatidaAsterinidae

(Lütken, 1860)

Figure 7a–e

Asterina minuta Gray, 1840: 289.
Asteriscus folium Lütken, 1860: 60–61.
Asterina folium A. Agassiz, 1877: 106, pl. 14, figs 7–9.
Asterinides folium Verrill, 1913: 479; Brito 1962: 3; 1968: 17, pl. 7, figs 2–3; 1971: 262. Tommasi 1970: 15, fig. 38. Oliveira et al. 2010: 3, fig. 2a.

###### Material examined.

Paraíba: 06°59'S; 34°47'W, 1spec., UFPB/ECH.572, 07.III.2006, 10m. Bahia: Camaçari, Guarajuba, Busca Vida Beach, 1spec., UFBA00685, 01.II.2006; 2spec., UFBA00983, I.2010, 23m; 1spec., UFBA01163, VII.2010, 25m; 2spec., UFBA01107, I.2010, 23m; Salvador, Todos os Santos Bay, 1spec., UFBA01107, 05.IV. 1997, 12m; Salvador: Itapuã, 12°57'28"S; 38°21'22"W, 1spec., UFBA00528, 19.XI.2007, 1m.

###### Type locality.

Saint Thomas, Virgin Islands (O’Loughlin 2002).

###### Description.

**Figure 7.**
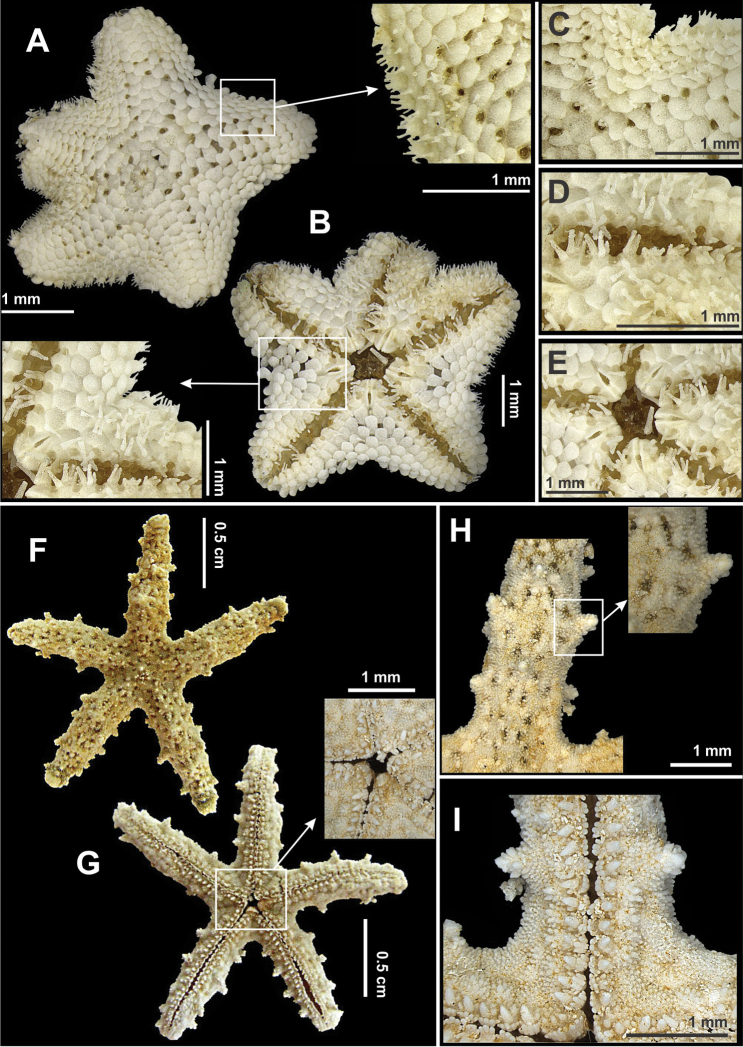
Some species of the order Valvatida recorded in northeastern Brazil. *Asterinides folium* **(A–E)** **A** Abactinal view, in detail the superomarginal figs **B** Actinal view, in detail actinal internidate area **C** Detail the abactinal intermediate are **D** Actinal view of the arm **E** Detail of the mouth; *Mithrodia clavigera* **(F–I)** **F** Abactinal view **G** Actinal view, in detail the mouth **H** Abactinal view of the arm, in detail a spine, and **I** Actinal view of the arm.

Body inflated, pentagonal (Fig. 7a, b). Five short arms (some specimens may have 4–6 arms). Abactinal figs imbricated, decreasing in size towards the margin of the body, with 2–4 short, hyaline, divergent spines, which have the extremity trifurcate (Fig. 7c). Between each of these figs there is a papula (Fig. 7c). Anus located in the center of the abactinal surface. Superomarginal figs similar to the remaining abactinal figs, but with more numerous and slightly longer spines, forming a dense bundle (Fig. 7a). Papulae restricted to the abactinal surface. Actinal figs similar to the abactinal figs, but a little bigger and longer, having 1–3 divergent spines, also similar to the dorsal ones, but slightly longer (Fig. 7b). Inferomarginal figs similar to the remaining actinal figs. Adambulacral figs with three thin, vitreous, elongate spines (~0.035 mm), having the tips trifurcate (Fig. 7d). Six thin, vitreous, elongate oral spines (~0.43 mm) (Fig. 7e).

**Colour.** Juvenile specimens vary from white to cream-coloured, larger juveniles are yellow to reddish, while adults are blue or greenish-blue (Hendler et al. 1995). Brito (1968) recorded a dark grayish-blue for 25 specimens from Trindade Island (ES).

###### Distribution.

Bermudas, Florida, Bahamas, Belize, Panama, and Brazil (Verrill 1915, Clark and Downey 1992, Hendler et al. 1995, Alvarado et al. 2008, Benavides-Serrato et al. 2011). In Brazil: BA, RJ, and Trindade Island (Brito 1962, 1968, 1971, Oliveira et al. 2010). In the presente study we record for the first time its presence in the State of Paraíba. Intertidal to 15 m in depth (Hendler et al. 1995).

###### Remarks.

Only two species of the genus *Asterinides* are known from the Atlantic Ocean, *Asterinides folium* and *Asterinides hartmeyeri* (Döderlein, 1910). The first is recorded for Bermudas and southern Brazil and the second only for the Caribbean region. According to Clark and Downey (1992) these species are partially sympatric, and for this reason have previously easily been confused with each other. In a revision of family Asterinidae based on molecular and morphological data, O’Loughlin and Waters (2004) transferred *Asterinides folium* and *Asterinides hartmeyeri* from the genus *Asterina* to the genus *Asterinides*, remarking on the morphological similarities between these two species. *Asterinides folium* differs from *Asterinides hartmeyeri* for having bigger papular pores, 6 to 7 series of actinal figs and abactinal figs arranged into two rows. A broad discussion of the main differences between these two species is given by Clark and Downey (1992). According to Hendler et al. (1995), this species rarely reaches 2.5 cm in diameter. Specimens examined in this study had a larger ray (R, maximum of 7.76 mm) and differed from those described by Hendler (op cit.) for not having narrow radial areas inflated, having instead the abactinal surface completely inflated.

###### Ecological notes.

This species lives in association with coral reefs, being found particularly under rocks or corals of the reef flat (Hendler et al. 1995). The specimen from Paraíba recorded in this study was found associated with rhodolite beds at 10 m depth. Although the species is reported from several localities, is has never been found in large numbers (Hendler et al. 1995, Benavides-Serrato et al. 2011). Only Brito (1971) observed this species to be relatively abundant mainly under rocks at Trindade Island.

#### Family Mithrodiidae Viguier, 1878

##### Mithrodia clavigera

Taxon classificationAnimaliaValvatidaMithrodiidae

(Lamarck, 1816)

Figure 7f–i

Asterias clavigera Lamarck, 1816: 562.
Mithrodia clavigera Verrill, 1870: 289.
Mithrodia spinulosa Gray, 1840: 288.
Ophidiaster echinulatus Müller & Troschel, 1842: 32.
Echinaster echinulatus von Martens, 1866: 59.
Mithrodia clavigera Perrier, 1875: 378.
Mithrodia victoriae Bell, 1882: 123, pl. 6, fig. 2. Brito 1962: 3; 1968: 16. Tommasi 1970: 19, fig. 55.

###### Material examined.

Paraíba: 7°04'S; 34°41'W, 1 spec., UFPB.ECH.880, 17.II.1981, 26m.

###### Type locality.

Unknown (Clark and Downey 1992).

###### Description.

Disk small (Fig. 7f). Five cylindrical and narrow arms (~2.52 mm) (Fig. 7f, g). Abactinal and actinal surfaces granulose (Fig. 7h). Skeleton formed by polygonal primary figs (usually hexagonal) that are widely spaced and united by secondary figs of rectangular shape, forming a reticulum. Some carinal and adradial figs with a long, narrow, and blunt spine (~0.77 mm). Papula large and single, found between the abactinal figs. Papulae restricted to abactinal surface. Granules covering body and spines small and with spinous tip. Granules from base of spines larger than at other localities. One conical and elongate subambulacral spine (~0.32 mm), forming a well defined row at base of ambulacral groove (Fig. 7i). Four slightly flattened adambulacral spines, the median ones being the largest. Eight short and rectangular oral spines, of which the median ones are largest (Fig. 7g).

**Colour.** Arms banded with dark brown or red lines, unusually green (Clark and Downey 1992). Specimens preserved in alcohol become uniformly white.

###### Distribution.

Mexico, Cuba, Nicaragua, Brazil, Indo-Pacific (except Hawaii) (Hayashi 1940, Abreu-Pérez et al. 2005, Alvarado et al. 2008). In Brazil: ES (Vitória Banks) (Brito 1968, Clark and Downey 1992). In this study we provide the first record for northeastern Brazil. From 24 to 71m in depth (Clark and Downey 1992).

###### Remarks.

*Mithrodia clavigera* is the only species of the family Mithrodiidae recorded for the Western Atlantic. For some time, two species were considered present: *Mithrodia clavigera* and *Mithrodia victoriae*. The later species was described by Bell (1882), based on two small specimens from submerged banks of Vitória (Victoria Bank) (Espírito Santo, Brazil). Since its description the validity of *Mithrodia victoriae* was questioned, and the distinction between these two species was discussed by Engel et al. (1948) and Pope and Rowe (1977). These authors concluded that the species should be synonymized but, due to the lack of material, this action was not formally carried out. Clark and Downey (1992) analised a large number of individuals of several sizes and agreed with the observations of Engel et al. (op. cit.) and Pope and Rowe (op. cit.), considering the two species to be synonyms. The individual we analised is juvenile (R = 9.80 mm) and corresponds to the characterization of juvenile specimens by Engel et al. (1948). These authors provide details on the aspect of the pedicellariae of *Mithrodia clavigera*, but pedicellariae were not found in our specimen.

###### Ecological notes.

This species lives on hard substrates covered by incrusting organisms and in reef gravel (Abreu-Pérez et al. 2005). For Paraíba it was recorded associated with rhodolith banks. According to Guille et al. (1986) this species is more active during the night.

#### Family Oreasteridae Fisher, 1911

##### Oreaster reticulatus

Taxon classificationAnimaliaValvatidaOreasteridae

(Linnaeus, 1758)

Figure 8a–g , 12c

Asterias gigas Linnaeus, 1753: 114.
Asterias reticulata Linnaeus, 1758: 661.
Pentaceros reticulatus Gray, 1840: 276.
Oreaster reticulatus Linnaeus, 1758. Tommasi 1958: 16–17, pl. 3, fig. 2; 1970: 10–11, fig. 31. Brito 1962: 3; 1968: 5–6, pl. 2, figs 1–3. Lima-Verde 1969: 11. Fernandes et al. 2002: 422. Magalhães et al. 2005: 63. Ventura et al. 2007: 238. Manso et al. 2008: 185, fig. 8c, d, e. Xavier 2010: 75. Alves et al. 2010: 757. Miranda et al. 2012: 143, 144.
Oreaster aculeatus Müller & Troschel, 1842: 50.
Oreaster lapidarius Grube, 1857: 342.
Oreaster tuberosus Möbius, 1859: 6.
Oreaster gigas Lütken, 1860: 64–75.
Oreaster reticulatus var. bermudensis H.L. Clark, 1942: 372, figs 1–2.

###### Material examined.

Ceará: off Fortaleza, 1spec., UFPB/ECH.1255, Geomar XXIV, V.1985. Paraíba: 1spec., UFPB/ECH.1579, 26.X.1980; Cabedelo, Farol de Cabedelo Reef, UFPB/ECH.1254, 22.I.1981, 26m; 1spec., UFPB/ECH.1588, 26.X.1980; 6°39'S; 34°49'W, 1spec., UFPB/ECH.1575, 28.V.1981, 20m; 6°39'5"S; 34°46'W, 1spec., UFPB/ECH.1429, 1spec., 29.V.1981, 35m; 6°39´05"S; 34°49"W, 1spec., UFPB/ECH.1251, 28.V.1981, 20m; 6°39'05"S; 34°49'W, 1spec., UFPB/ECH.1578, 28.V.1981, 20m; 6°50'S; 34°47'W, 1spec., UFPB/ECH.1253, 11.V.1981, 18m; 6°52'S; 34°46'W, 1spec., UFPB/ECH.1872, 19.II.1981, 18m; 6°52'S; 34°48'W, 1spec., UFPB/ECH.1590, 04.II.1981, 10m; 6°52'S; 34°49'W, 1spec., UFPB/ECH.1593, 04.II.1981, 12m; 6°57'S; 34°41'W, 1spc., UFPB/ECH.1577, 12.II.1981, 26m; 7°01'S; 34°47'05"W, 2spec., UFPB/ECH.1252, 02.V.1981, 11m; 7°04'S; 34°41'W, 2spec., UFPB/ECH.1576, 17.II.1981, 22m; 07°04'24,4"S; 034°47'49"W, 1spec., UFPB/ECH.1871, 24.VI.2005, 6m; 7°07'S; 34°47'W, 1spec., UFPB/ECH.1430, 05.II.1981, 10m; 7°10'S; 34°38'W, 1spec., UFPB/ECH.1873, 26.03.1981, 25m; 7°13'S; 34°42'W, 1spec., UFPB/ECH.1574, 27.III.1981, 10m; 7°34'S; 34°39'W, 1spec., UFPB/ECH.1594, 1spec., 7°34'S; 34°39'W, Paraíba, 22.I.1981, 26m. Pernambuco: Itamaracá Island, 4spec., UFPB/ECH.1581, VIII.1980, 20m; Suape Beach, 1spec., UFPB/ECH.1580, I.1980.

###### Type locality.

‘Las Antillas Occidentales’ or ‘Spanish West Indies’ (Clark and Downey 1992).

###### Description.

**Figure 8.**
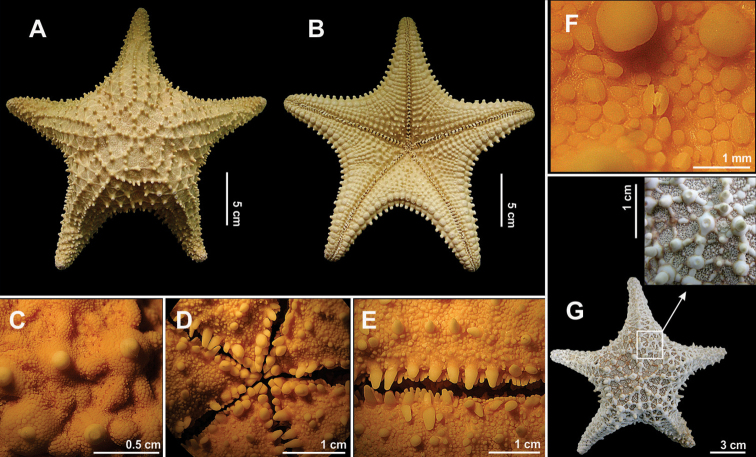
Some species of the order Valvatida recorded in northeastern Brazil. *Oreaster reticulatus* **(A–G)** **A** Abactinal view **B** Actinal view **C** Detail view da abactinal surface **D** Detail of the mouth **E** Actinal view of the arm **F** Detail of the bivalve pedicellariae, and **G** Skeleton, in detail its arrangement into a reticulum.

Body pentagonal. Disk high, inflated, concave on actinal side (Fig. 8a, b). Five short arms (Fig. 8a). Abactinal figs with one thick, short, blunt spine (~2.43 mm). Among these figs there occur papular areas, which are covered by small granules and bivalve pedicellariae (Fig. 8c). These granules cover the entire body, including the bases of the spines. Superomarginal figs with one short, thick and blunt spine (~3.38 mm), determining the margin of the body. Inferomarginal figs similar to superomarginal figs, with one or two small and thick spines. Papular areas restricted to abactinal surface. Actinal surface granulose, with a great number of pedicellariae, especially in areas near the mouth and abulacral groove. Actinal figs with 1–2 short, conical, and blunt spines (~1.53 mm). Ambulacral figs with 5–6 short and flattened spines, of which the median spines are the largest (Fig. 8e). Short, conical, spines (~3.16 mm) form a well defined row of spines on the margins of the ambulacral grooves. Four short, thick, blunt oral spines (Fig. 8d). Sessile bivalve pedicellariae distributed over entire body of animal (Fig. 8f). Skeleton formed by conical, abactinal figs interconnected by secondarily elongated and widened figs, arranged into a reticulum (Fig. 8g).

**Colour.** According to Hendler et al. (1995) and Verrill (1915) the colour pattern of this species is very variable, even among individuals from a same population. Along the Brazilian coast the most common colour is orange or brownish red. Yet juvenile individuals differ significantly from adults. According to Benavides-Serrato et al. (2011) and Hendler et al. (1995) the aboral surface of juveniles is frequently olive-green and usually presents green-grey or coffe-coloured spots. In the adults, on the other hand, this colour is orange with lighter or darker tubercles on the disk and arms. The oral surface in both stages is beige or cream-coloured.

###### Distribution.

North and South Carolina, the Bermudas, the Bahamas, Belize, Guatemala, Honduras, Cuba, Nicaragua, Costa Rica, Venezuela, Brazil, and Cabo Verde (Tommasi 1970, Walenkamp 1976, Hendler et al. 1995, Ventura et al. 2007, Alvarado et al. 2008, del Valle García et al. 2008). In Brazil: MA, CE, PB, PE, AL, BA, RJ, SP, and SC, incluinding Abrolhos and Trindade Island (Rathbun 1879, Verrill 1915, Clark 1942, Tommasi 1958, 1970, Brito 1962, 1968, Lima-Verde 1969, Fernandes et al. 2002, Magalhães et al. 2005, Xavier 2010, Miranda et al. 2012). In this study we record the species for the first time in the States of Rio Grande do Norte, and Alagoas. From 0 to 800 m in depth, being most abundant up to 50 m.

###### Remarks.

Only two species of the genus *Oreaster* are known for the Atlantic Ocean, *Oreaster clavatus* and *Oreaster reticulatus*. The first is known only from the Island of Cape Verde, São Thomé and the Gulf of Guinea. The second, occurs widely throughout the West Atlantic, from North Carolina to the south of Brazil (Clark and Downey 1992), its known southern limit being located in the State of Santa Catarina. *Oreaster reticulatus* differs from its congeneric *Oreaster clavatus* for presenting an inflated body and abactinal figs with tubercules or spines. Clark (1942) described the variety Oreaster reticulatus var. bermudensis on the basis of the irregular placement of spines and papulae on the abactinal surface and of the presence of only one spine on the actinal figs. However, these characters also occur in other specimens from the Atlantic and thus do not sustain the name. According to Hendler et al. (1995) the species may attain a disk diameter of up to 500 mm. We observed morphological variations in both juveniles and adults, but were not able to correlate these with colour patterns in this preserved material.

###### Ecological notes.

The species lives in shallow reef environments with calm water, coastal lagoons, seagrass beds (*Thalassia*, *Halodule* and *Syringodium*), and mangrove channels (Benavides-Serrato et al. 2011). In this study the species was recorded in rhodolite beds and coastal reefs below 6 m. According to Verrill (1915) *Oreaster reticulatus* was the most abundant species in the States of Bahia and Pernambuco. Presently it is difficult to find along the northeastern coast of Brazil, and is listed as vulnerable to extinction (Machado et al. 2008). This is an omnivorous species, feeding mainly on microorganisms from organic matter associated with the sand of seagrass beds and algal substrates. However, it is also an opportunistic predator of echinoderms, such as *Tripneustes ventricosus* (Lamarck, 1816) and *Meoma ventricosa ventricosa* (Lamark, 1816), as well as of individuals belonging to its own species and to a large variety of sponges (Hendler et al. 1995). The only known predator of adults belonging to this species is the gastropod *Charonia variegata* (Lamarck, 1816), while young individuals are known to be eaten by a great variety of fishes (Scheibling 1980).

### Order Velatida Perrier, 1884

#### Family Pterasteridae Perrier, 1875

##### Calyptraster coa

Taxon classificationAnimaliaVelatidaPterasteridae

Sladen, 1882

Figure 9a–h

Calyptraster coa Sladen, 1882: 207. Tommasi 1970: 13.
Calyptraster personatus Madsen, 1947: 3–7, figs 1–2.

###### Material examined.

MZUSP (without registration number), 1spec., W Besnardi, dredging 5142. MZUSP (without registration number), 2spec., W Besnardi, dredging 5363.

###### Type locality.

Recife, Pernambuco, Brazil (Clark and Downey 1992).

###### Description.

**Figure 9.**
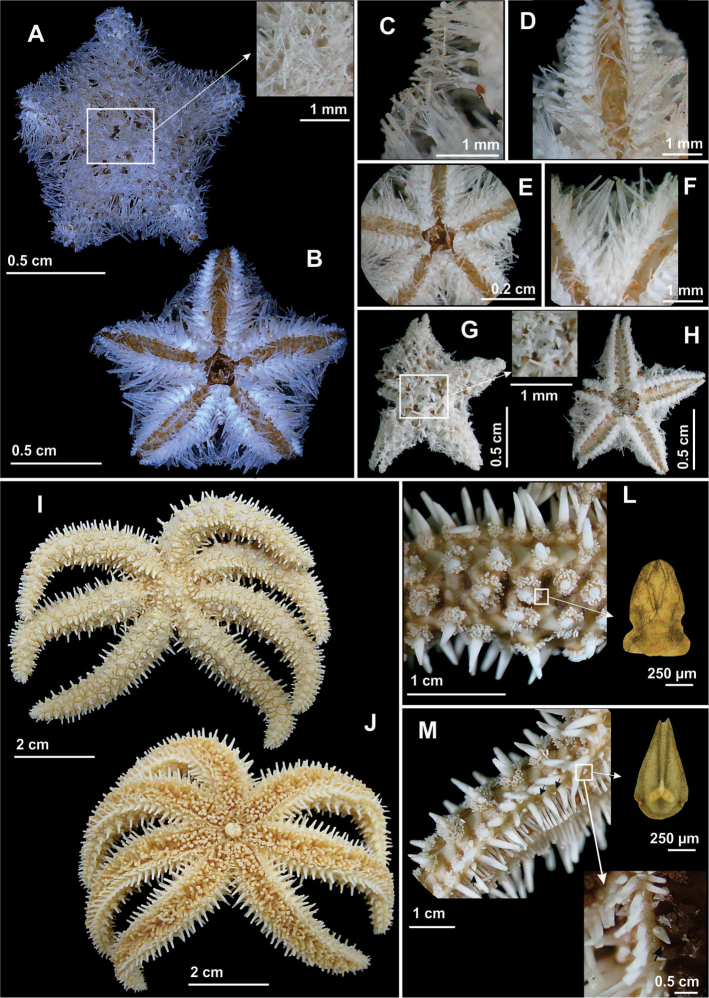
Some species of the order Velatida **(A–H)** and Forcipulatida **(I–M)** recorded in northeastern Brazil. *Calyptraster coa* **(A–H)** **A** Abactinal view, in detail the supradorsal membrane **B** Actinal view **C** Detail of the paxillae with long peduncles **D** Actinal view of the arm **E** Detail of the mouth **F** Detail of the actinal intermediate area **G** Abactinal view, in detail oscular valves **H** Actinal view; *Coscinasterias tenuispina* **(I–M)** **I** Abactinal view **J** Actinal view **L** Abactinal view of the arm, in detail the bivalve pedicallariae (optical microscopic image), and **M** Lateral view of the arms, in detail the bivalve pedicellariae (optical microscopic image).

Body pentagonal (Fig. 9a, b, g, h). Five short arms. Supradorsal membrane thin and transparent (Fig. 9a). Spiracules moderately large, numerous, irregularly distributed. Oscule large, surrounded by long oscular valves with an enlarged extremity (Fig. 9g). Abactinal surface with paxillae. Paxillae with long peduncles and a crown of 5 to 6 long and vitreous spinelets (Fig. 9c). Skeletal figs narrow, long and vitreous, forming a reticulum. Actinal surface slightly concave. Oral spines long and vitreous, the lateral ones being longer and thicker (Fig. 9e). Adambulacral figs with three or four spines (Fig. 9d).

**Colour.** From light brown to hey-coloured (Sladen 1889).

###### Distribution.

The Bahamas, Florida, and Brazil (Clark and Downey 1992). In Brazil: PE (Tommasi 1970; Clark and Downey 1992). From 260 to 933 m in depth (Clark and Downey 1992).

###### Remarks.

The genus *Calyptraster* presently contains five species (Mah 2013), four of which occur in the Western Atlantic: *Calyptraster coa*, *Calyptraster personatus* (Perrier, 1885), *Calyptraster tenuissimus* Bernasconi, 1966 and *Calyptraster vitreus* Bernasconi, 1972. The first two have a similar geographical distribution, occurring from the Bahamas to Brazil and Colombia, respectively, while the last two are restricted to the coast of Argentina. According to Clark and Downey (1992), this genus is problematic, and the distinctions between *Calyptraster coa* and *Calyptraster personatus* remain to be better established. Walenkamp (1979) provides good descriptions and discusses the main morphological diferences between these species. In the phylogenetic analysis of the family Pterasteridae by Villier et al. (2004), it is concluded that *Calyptraster* representes a monophyletic genus. *Calyptraster coa* differs from *Calyptraster personatus* for having conical suboral spines, an osculum surrounded by valves with enlarged extremities, and for being distributed no deeper than 1000 m. Specimens observed in this study were all juveniles. Smaller specimens tend to have longer arms and a more flattened body. Our material was badly preserved in general, not permitting a detailed description of their morphological characters.

###### Ecological notes.

This species is quite rare, from deep waters, with little known of its biology and ecology. The species was collected in bottoms containing red mud (Sladen 1889).

#### Family Ophidiasteridae Verril, 1870

##### Linckia guildingi

Taxon classificationAnimaliaVelatidaOphidiasteridae

Gray, 1840

Figure 10a–e , 12d

Linckia guildingii Gray, 1840: 285. Tommasi 1958: 17. Brito 1962: 3; 1968: 4–5, pl. 1, fig. 3; 1971: 262. Lima-Verde 1969: 11. Tommasi and Aron 1988: 3. Tommasi et al. 1988: 6. Fernandes et al. 2002: 422. Gondim et al. 2008: 154.
Linckia pacifica Gray, 1840: 285.
Ophidiaster ornithopus Müller & Troschel, 1842: 31
Ophidiaster ehrenbergi Müller & Troschel, 1842: 31
Linckia ornithopus Verrill, 1867: 344.
Linckia nicobarica Lütken, 1872: 265.
Linckia ehrenbergi Loriol, 1885: 31.
Linckia guildingi Verrill, 1907: 325. Miranda et al. 2012: 144.

###### Material examined.

Paraíba: 6°29'S; 34°48'W, 1spec., UFPB/ECH.1235, 04.VI.1981, 30m; 6°29'S; 34°51'W, 1spec., UFPB/ECH.1244, 05.VI.1981, 22m; 6°33'S; 34°51'W, 1spec., UFPB/ECH.1243, 04.VI.1981, 20m; 7°01'S; 34°30'W, 1spec., UFPB/ECH.1856, 13.II.1981, 26m; 7°01'S; 34°30'W, 3spec., UFPB/ECH.1238, 13.II.1981, 26m; 7°04'S; 34°44'W, 1spec., UFPB/ECH.1237, 16.II.1981, 16m; 7°12'5"S; 34°36'W, 1spec., UFPB/ECH.1241, 01.IV.1981, 26m; 7°28'S; 34°34'W, 1spec., UFPB/ECH.1242, 06.V.1981, 30m; João Pessoa, Cabo Branco Beach, 1spec., UFPB/ECH.1159, 19.II.2003. 1spec., UFPB/ECH.1160, 04.XI.2006, 1spec., UFPB/ECH.1161, 06.X.1979; 3spec., UFPB/ECH.1236, 25.X.2007, 1spec., UFPB/ECH.1245, 16.V.2007, 1spec., UFPB/ECH.1246, 19.IV.2005; 1spec., UFPB/ECH.1247, 08.IX.2006, 2spec., UFPB/ECH.1248, 25.X.2007, 1spec., UFPB/ECH.1250, 03.VII.2004, 1spec., UFPB/ECH.1479, 13.XI.2008, 1spec., UFPB/ECH.1502, 08.II.2009, 1spec., UFPB/ECH.1867, III.2007. Pernambuco: Paulista, Pau Amarelo reef, 1spec., UFPB/ECH.1845, 08.XI.1982. Alagoas: Paripueira, Paripueira Beach, 2spec., UFPB/ECH.1851, 01.II.1983; Maceió, Ponta Verde Beach, 1spec., UFSITAB-199, I.2007, 1spec., UFSITAB-200, I.2007; Marechal Deodoro, Francês Beach, 5spec., UFPB/ECH.1855, 29.I.1983, 2spec., UFPB/ECH.1847, 20.XII.1984, 1spec., UFPB/ECH.1849, 19.II.1985, 2spec., UFPB/ECH.1857, 19.II.2011. Bahia: Salvador, Itapoã Beach, 1spec., UFPB/ECH.1848, 21.XII.1984; Itaparica, Pedrão, 4spec., UFPB/ECH.1853, 18.IX.1982; Itaparica, Barra Grande, 1spec., UFPB/ECH.1854, 17.IX.1982; Santa Cruz da Cabrália, Ponta da Coroa Vermelha, 5spec., UFPB/ECH.1846, 15.X.1982; Santa Cruz da Cabrália, Ponta do Mutá reef, 5spec., UFPB/ECH.1850, 16.X.1982; Porto Seguro, Ponta Grande reef, 5spec., UFPB/ECH.1852, 15.X.1982.

###### Type-locality.

Saint Vincent, West Indies (Clark and Downey 1992).

###### Description.

**Figure 10.**
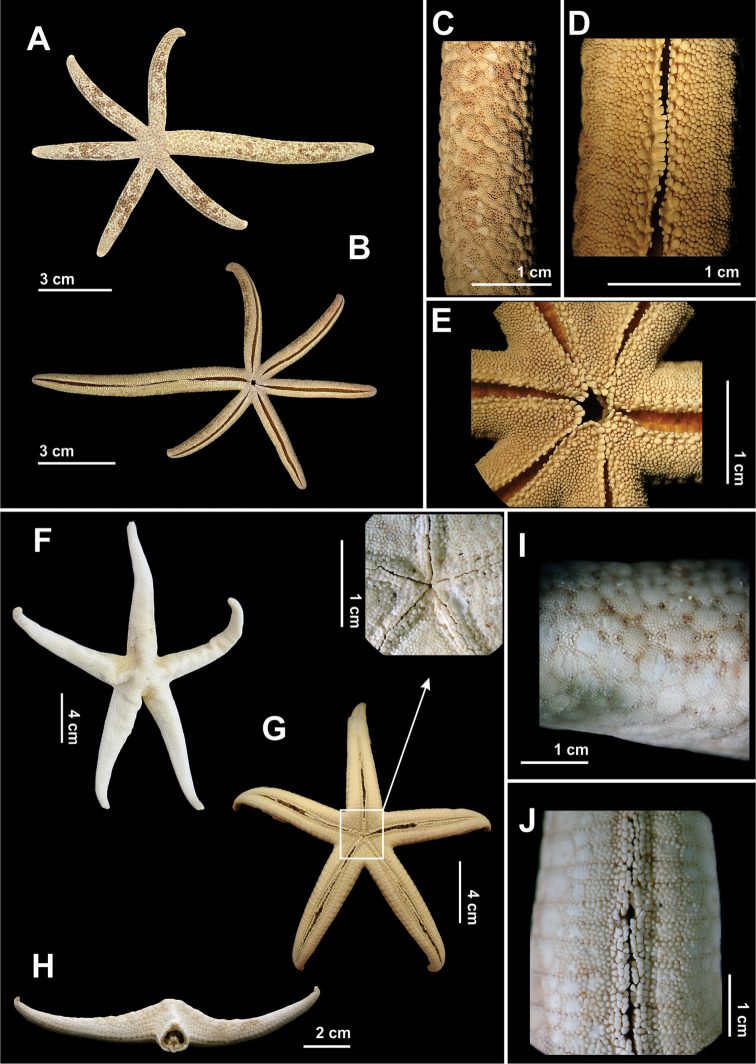
Some species of the order Velatida recorded in northeastern Brazil. *Linckia guildingi* **(A–E)** **A** Abactinal view **B** Actinal view **C** Abactinal view of the arm **D** Actinal view of the arm **E** Detail of the mouth; *Narcissia trigonaria* **(F–J)** **F** Abactinal view **G** Actinal view, in detail the mouth **H** Lateral view **I** Abactinal view of the arm, and **J** Actinal view of the arm.

Disk small. From four to six long, thin, cylindrical arms (Fig. 10a, b). Abactinal and actinal surfaces granuliform. Two or more madreporites. Abactinal figs rounded, inflated, irregularly arranged. Among these figs there are papular areas with 5–23 pores (Fig. 10c). Papular areas restricted to abactinal surface. Superomarginal and inferomarginal figs similar and uniform in size, separated by a row of papular areas. Actinal figs forming 2–3 series, which extend to the tip of the arms and are covered by granules a little larger than those on abactinal surface. Adambulacral figs with two short, blunt, parallel spines, one being much larger than the other (Fig. 10d). Behind these there is one wide, thick, blunt, ambulacral spine. Oral spines slightly longer (~0.68 mm) than remaining spines, having their tips rounded (Fig. 10e). Ocular figs also granulose.

**Colour.** Extremely variable, usually juveniles and adults having different colours (Hendler et al. 1995). Juvenile individuals have brown, red or violet spots, while adults are uniformly reddish-brown, yellowish-brown, violet, or olive-green (Brito 1960, Hendler et al. 1995, Benavides-Serrato et al. 2011). In the Brazilian material the most common colour observed is brown and yellow.

###### Distribution.

Tropicopolitan (Tommasi 1970, Alvarado et al. 2008). It also occurs throghout the tropical Indo-West Pacific. In Brazil: PB, PE, AL, BA, RJ, and SP, including Abrolhos and Trindade Island (Rathbun 1879, Verrill 1915, Bernasconi 1955, Tommasi 1958, Brito 1960, 1962, 1968, 1971, Lima-Verde 1969, Tommasi and Aron 1988, Fernandes et al. 2002, Magalhães et al. 2005, Gondim et al. 2008, Miranda et al. 2012). From 0 to 298 m in depth (Clark and Downey 1992).

###### Remarks.

Two species of the genus *Linckia* are known for the Brazilian coast, *Linckia guildingi* and *Linckia nodosa* Perrier, 1875, the latter recorded only for south and southeastern Brazil. Tommasi and Aron (1988) cite *Ophidiaster guildingi* Gray, 1840 for southeast Bahia, a locality we were not able to confirm. *Linckia guildingi* differs from *Linckia nodosa* for having small triangular abactinal figs, 18–30 pores per papular area, and two subambulacral spines. Juvenile individuals of *Linckia guildingi* may be confused with *Ophidiaster guildingi* Gray, 1840, which have the same colour and occupy the same habitat (Hendler et al. 1995). *Ophidiaster guildingi* differs from *Linckia guildingi* for having less than 15 pores per papular area. Although we observed both juvenile and adult individuals, no morphological variations were noted.

###### Ecological notes.

This species lives in environments with consolidated substrates or sand banks among reefs (Machado et al. 2008). It has cryptic habits, being found mostly under rocks. Possibly *Linckia guildingi* uses the film of microorganisms adhered to the substrate as food (Hendler et al. 1995; Machado et al. 2008). According to Brito (1971) this species is abundant along the northeastern coast of Brazil. However, its populations are becoming reduced, especially in southeastern Brazil. The species is presently included among the species vulnerable to extinction (Machado et al. 2008). *Linckia guildingi* is known for its strong propensity to autotomize and its capacity to regenerate. Specimens with four, six or seven arms are common (Tommasi 1958).

##### Narcissia trigonaria

Taxon classificationAnimaliaVelatidaOphidiasteridae

Sladen, 1889

Figure 10f–j

Narcissia trigonaria Sladen, 1889: 414, pl. 65, figs 5–8. Tommasi 1966: 244; 1970: 9, fig. 26. Brito 1960: 5, pl. 1, figs 4–5; 1962: 3; 1968: 5. Tommasi and Aron 1988: 3. Tommasi et al. 1988: 6. Miranda et al. 2012: 144.
Narcissia trigonaria var. helenae Mortensen, 1933: 429.

###### Material examined.

Alagoas: Lagoa Azeda, Jequidá da Praia, 1spec., MNRJ (no registration number), 22.VI.2002. Bahia: Salvador, north coast, 1spec., UFBA00570, 2003; Salvador, Porto da Barra, 1spec., UFBA00929, II.2008, 23m; Salvador, Barra Beach, 1spec., UFBA00962, X.2008; Itaparica, Ponta de Areia, 12°52'S; 38°40'W, 1spec., UFBA00469; Camaçari, Guarajuba, 1spec., UFBA00190, VII.2005, 23m; Camaçari, Guarajuba, Busca Vida Beach, 2spec., UFBA00042, 04.VI.1994, 1spec., UFBA01043, VII.2008, 26m, 1spec., UFBA01089, VII.2008, 32m.

###### Type-locality.

Bahia, Brazil (Clark and Downey 1992).

###### Description.

Disk high and pyramidal (Fig. 10f, h). Five long and triangular arms in transversal section (Fig. 10f, h). Abactinal and actinal surfaces granulose (Fig. 10i). Abactinal figs rounded, placed in irregular rows, covered by flattened and polygonal granules. Among these figs are papular areas with up to three papulae. Superomarginal figs short, wide (~2.69 mm), granulose and visible only laterally. Papular areas restricted to abactinal surface. Inferomarginal and superomarginal figs similar. Actinal figs slightly rectangular and granulose, these granules being bigger and taller than the dorsal granules. Actino-lateral figs with two rows of spines, the outer series with 4–5 short and blunt spines. The inner row is formed by 3–4 large, flattened spines, being longer than the outer row. Adambulacral figs with 3–4 series of flattened and prismatic spines, of which the innermost are the largest (Fig. 10j).

**Colour.** Live specimens are cream-coloured with red spots (Benavides-Serrato et al. 2011).

###### Distribution.

North Carolina, Florida, Panama, Colombia, and Brazil (Tommasi 1970; Clark and Downey 1992; Alvarado et al. 2008; Benavides-Serrato et al. 2011). In Brazil: AL, BA, and RJ (Verrill 1915, Brito 1960, 1962, Tommasi 1970, Tommasi and Aron 1988, Miranda et al. 2012). From 5 to 91 m in depth (Tommasi 1970, Clark and Downey 1992).

###### Remarks.

*Narcissia trigonaria* is a well established species, with a small list of synonyms and little morphological variation. It differs from *Narcissia canariensis* (d’Orbigny, 1839) for having subambulacral spines arranged into three series and paired papulae. Downey (1973) records the sugar-tongs type of pedicellariae among the carinal figs of *Narcissia trigonaria*. However, we did not observe this type of pedicellaria. Walenkamp (1976) gives an excellent discussion on the presence or absence of pedicellariae and on small morphological variations found in his material. He emphasizes the great morphological differences existing between juvenile and adult specimens. In general, very juvenile individuals have shorter and wider arms. These tend to become thinner and longer during ontogenetic development.

###### Ecological notes.

The species lives in consolidated substrates, either rocks or coral (Machado et al. 2008). Presently it is considered vulnerable to extinction along the Brazilian littoral. The main causes of its populational decline are the effects of pollutants and its illegal and indiscriminate collecting for aquarium rearing (Machado et al. 2008).

### Order Spinulosida Perrier, 1884

#### Family Echinasteridae Verril, 1867

##### Echinaster (Othilia) brasiliensis

Taxon classificationAnimaliaSpinulosidaEchinasteridae

Müller & Troschel, 1842

Figure 11a–e

Echinaster brasiliensis Müller & Troschel, 1842: 22. Tommasi 1958: 22–23, pl. 4, fig. 3; 1970: 17, figs 44–45. Brito 1962: 3; 1968: 13–14, pl. 6, fig. 6. Carrera-Rodriguez and Tommasi 1977: 103–104. Tommasi and Aron 1987: 3. Tommasi et al. 1988: 6. Ávila-Pires 1983: 440–442, figs 8–9. Fernandes et al. 2002: 422. Netto 2006: 30–32, pl. 5a. Alves et al. 2010: 758. Miranda et al. 2012: 144.
Echinaster braziliensis Verrill, 1915: 41–42, pl. 26, fig. 1.
Echinaster antonioensis Bernasconi, 1955: 72–73, pl. 6, figs 1–2. Tommasi 1958: 22, pl. 4, fig. 2. Brito 1968: 15.
Echinaster sentus Bernasconi, 1956: 136–137, pl. 4, fig. 3. Tommasi 1958: 23–24, pl. 4, fig. 4; 1970: 17–18, fig. 46 a 48. Brito 1968: 14, pl. 6, figs 3–4.
Echinaster spinulosus Bernasconi, 1956: 138–139, pl. 4, fig.4. Tommasi 1958: 21–22, pl. 4, fig. 1. Brito 1968: 14, pl. 6, fig. 1–2.
Echinaster densispinulosus Tommasi, 1970: 18–19, figs 49–51.
Echinaster nudus Tommasi, 1970: 18–19, figs 52–54. Gondim et al. 2008: 154.
Echinaster (Othilia) brasiliensis Clark & Downey, 1992: 21–22, pl. 4a. Hopkins et al. 2003: 98–100. Machado et al. 2008: 182–183. Lima and Fernandes 2009: 59. Xavier 2010: 75.

###### Material examined.

Rio Grande do Norte: Macau, Diogo Lopes, 1spec., UFPB/ECH.869, 09.XI.2007, 1spec., UFPB/ECH.872, 09.XI.2008, 1spec., UFPB/ECH.1426, 09.XI.2007. Paraíba: 7°01'02"S; 34°47'55"W, 1spec., UFPB/ECH.571, 03.VI.2003; 7°03'50"S; 34°47'19"W, 1spec., UFPB/ECH.569, 21.III.2006; Cabedelo, Farol de Cabedelo reef, 1spec., UFPB/ECH.729, 26.X.1980; Cabedelo, Areia Vermelha reef, 1spec., UFPB/ECH.1465, 22.II.2008; João Pessoa, Cabo Branco Beach, 2spec., UFPB/ECH.138, 04.XI.2006; João Pessoa, Seixas reef, 1spec., UFPB/ECH.1183, 22.XII.2007. Bahia: Santo Amaro, Cabuçu Beach, 3spec., UFPB/ECH.718, 19.IX.1987.

###### Type-locality.

Ubatuta, São Paulo, Brazil (Walenkamp 1976).

###### Description.

**Figure 11.**
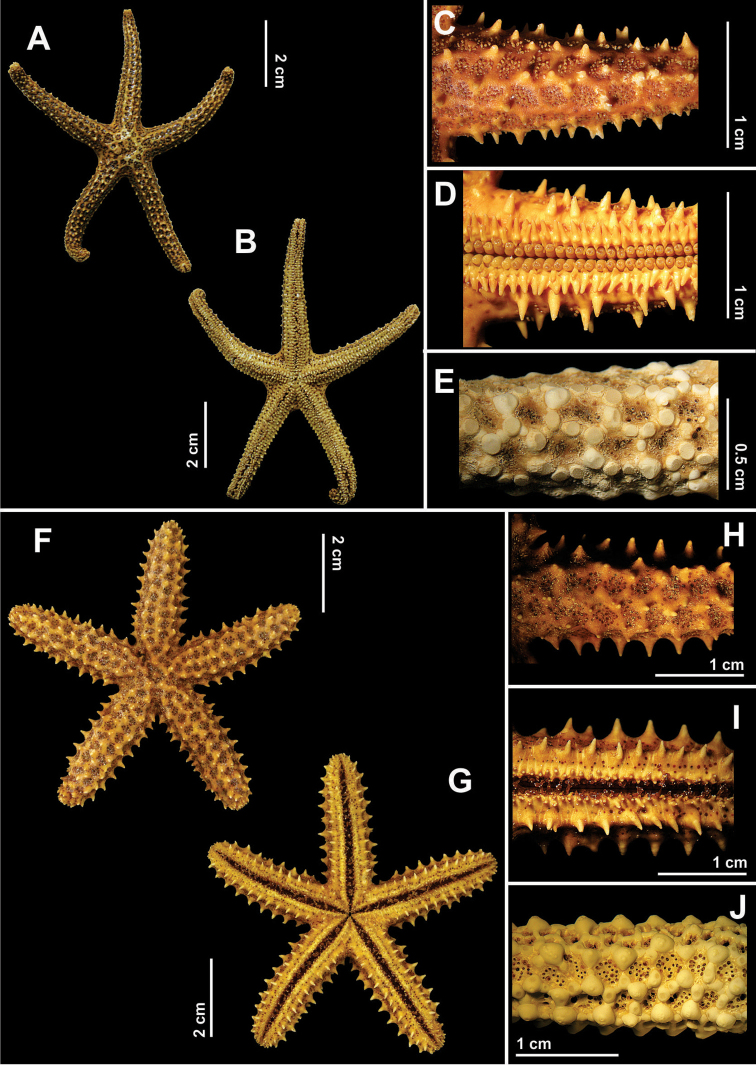
Some species of the order Spinulosida recorded in northeastern Brazil. Echinaster (Othilia) brasiliensis **(A–E)** **A** Abactinal view **B** Actinal view **C** Abactinal view of the arm **D** Actinal view of the arm **E** Arranjo do endoqesquelto do braço; Echinaster (Othilia) echinophorus **(F–J)** **F** Abactinal view **G** Actinal view **H** Abactinal view of the arm **I** Actinal view of the arm; and **H** Arranjo do endoesqueleto do braço.

Disk small (Fig. 11a). Body concave on abactinal surface, plane on actinal surface. Five long-narrow to short-thick arms (usually decreasing rapidly in thickness towards tips) (Fig. 11a, b). Abactinal figs flattened or slightly mammiform (Fig. 11e), forming a reticulum and bearing a short, conical spine (Fig. 11c). Abactinal figs separated by small, elongated, secondary figs. Anus normally placed within the central pentagon of the disk and surrounded by 4–6 spines. Madreporite circular, with numerous small granules and irregular sulci. Superomarginal figs more granulose than inferomarginal figs. Papular areas more numerous on dorsal and lateral regions of arms (Fig. 11c). Adambulacral figs with three spines, the inner one being rudimentary (Fig. 11d). The two outer spines are subequal and larger than the remaining spine.

**Colour.** Quite variable, being light brown, reddish-brown, dark red or even yellowish red (Gray et al. 1968; Benavides-Serrato et al. 2011).

###### Distribution.

Florida, Cuba, Honduras, Panama, Colombia, Brazil, and Argentina (Tommasi 1958, Alvarado et al. 2008, Benavides-Serrato et al. 2011). In Brazil: PB, PE, AL, BA, ES, SP, RJ, SC, and RS (Verrill 1915, Tommasi 1958, Carrera-Rodriguez and Tommasi 1977, Fernandes et al. 2002, Magalhães et al. 2005, Gondim et al. 2008, Xavier 2010, Miranda et al. 2012). This paper provides the first record for the State of Rio Grande do Norte. From 1 to 360 m in depth (Benavides-Serrato et al. 2011).

###### Remarks.

Many characters of Echinaster (Othilia) brasiliensis and other species of the genus present great plasticity, with the consequence that the taxonomy of the genus cannot be considered well resolved. Echinaster (Othilia) brasiliensis differs from Echinaster (Othilia) echinophorus for presenting a larger number of rows of dorsal longitudinal spines (9–15) and actinal figs not mammiform. Echinaster (Othilia) sentus differs for having more numerous and shorter dorsal spines and for having mammiform abactinal figs. Walenkamp (1976) pointed out some variations observed in specimens from Suriname, such as: number of adambulacral spines (from 2 to 5), number of spines per dorsal longitudinal row (from 1 to 15), and size of the dorsal papular areas. In our study, as well as the large plasticity in the number of dorsal longitudinal spines (from 7 to 13), a character used as diagnotic for the genus, the number of spines surrounding the anus and the shape of the arms also proved to be quite variable. However, these morphological variations do not seem to be related to ontogenetic stages. Despite both adults and juveniles being present in our material, these variations occurred among specimens of a same size class. According to Machado et al. (2008), the most common shape of Echinaster (Othilia) brasiliensis presents narrow and elongate arms, while a smaller proportion of individuals have short and thick arms and less numerous spines. Tommasi (1970) synonymized Echinaster (Othilia) antonioensis De Loriol, 1904 with Echinaster (Othilia) brasiliensis, though without providing further details. According to him (Tommasi *op. cit.*) the characters used by De Loriol to distinguish the two species are all dependent on fixation mode or represent highly variable characters. An excelent discussion on the synonyms of the several species of Echinaster (Othilia) brasiliensis is found in Clark and Downey (1992). Avila-Pires (1983) proposed the presence of only two species of *Echinaster* for the Brazilian littoral, Echinaster (Othilia) echinophorus being restricted to the northeastern coast and Echinaster (Othilia) brasiliensis to the south and southeastern coast. We disagree with this opinion, suggesting that Echinaster (Othilia) brasiliensis also occurs in northeastern Brazil. More taxonomic studies are clearly needed in order to better establish the interspecific limits between these two species.

###### Ecological notes.

This species lives in sand, substrates of sand with mud, and consolidated substrates, often associated with the bivalves *Mytillus* sp. and *Lithophaga* sp. (Penchaszadeh 1973), having also been observed in banks of *Thalassia* sp. (Benavides-Serrato et al. 2011). Echinaster (Othilia) brasiliensis is frequently found in intertidal regions or shallow waters, and may be strongly influenced by water salinity (Machado et al. 2008). In this study, the species was found mainly in reef environments and hypersaline mangrove areas, always together with Echinaster (Othilia) echinophorus. According to Machado et al. (2008), this species is common along the coast of Rio de Janeiro, being intensively collected by aquarists without futher control on their extraction and commercialization. Alves and Dias (2010) recorded its use for medical purposes (treatment of asthma). Echinaster (Othilia) brasiliensis is listed among the species vulnerable to extinction (Machado et al. 2008).

##### Echinaster (Othilia) echinophorus

Taxon classificationAnimaliaSpinulosidaEchinasteridae

(Lamarck, 1816)

**Figure 12.**
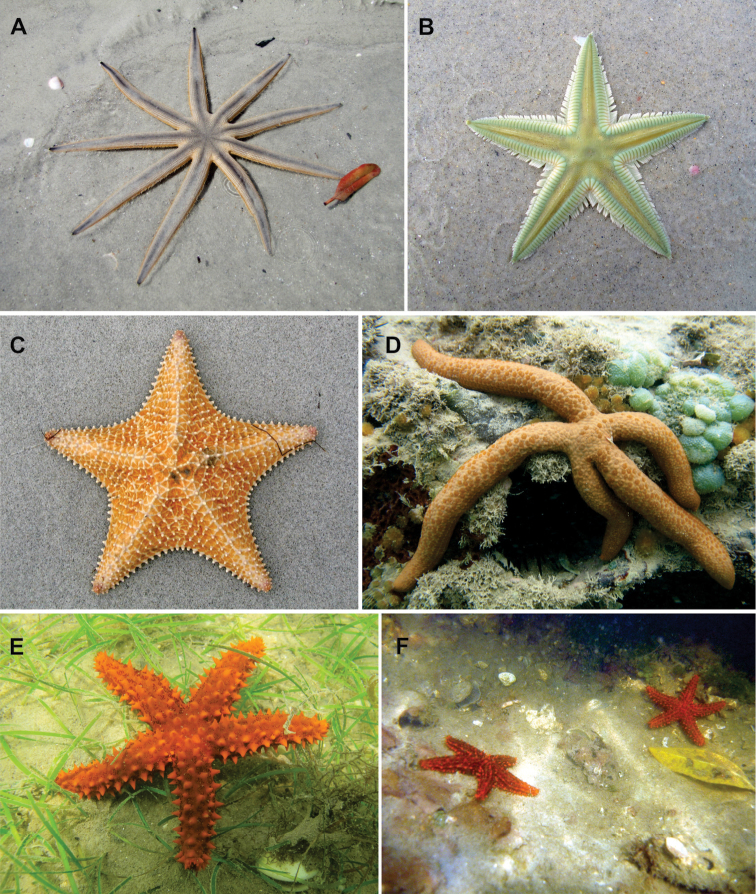
Some common starfishes in their natural habitat. **A** *Luidia senegalensis* in a sand beach **B** *Astropecten marginatus* in a hypersaline mangrove **C** *Oreaster reticulatus* in a sand beach **D** *Linckia guildingi* on coral reefs **E** Echinaster (Othilia) echinophorus in seagrass beds and **F** Echinaster (Othilia) echinophorus over the muddy bottom of a hypersaline mangrove. Photos: Thelma LP Dias.

Figures 11e–j , 12e–f

Asterias spinosa Retzius, 1805: 18.
Asterias echinophora Lamarck, 1816: 560.
Stellonia spinosa Nardo, 1834: 716.
Othilia spinosa Gray, 1840: 281.
Echinaster spinosus Müller & Troschel, 1842: 22.
Echinaster (Othilia) crassispina Verrill, 1868: 368.
Echinaster crassispinus Lütken, 1872: 285.
Echinaster echinophorus Perrier, 1875: 100–102. Brito 1962: 3. Lima-Verde 1969: 11. Avila-Pires 1983: 436–440, figs 6–7. Tommasi 1970: 16–17, figs 41–43. Tommasi and Aron 1988: 3. Fernandes et al. 2002: 422. Gondim et al. 2008: 155, fig. 3a. Alves et al. 2010: 757. Miranda et al. 2012: 144.
Othilia echinophora Fisher, 1919: 432.
Echinaster (Othilia) echinophorus Clark & Downey, 1992: 367–371. Magalhães et al. 2005: 63. Machado et al. 2008: 183–184. Lima and Fernandes 2009: 59. Gondim et al. 2011: 6, fig. 3e.

###### Material examined.

Rio Grande do Norte: Macau, Diogo Lopes, 4spec., UFPB/ECH.871, 09.XI.2007; Tubarão River, 1spec., UFPB/ECH.1905,14.XI.2009; Tubarão River Mangrove, 1spec., UFPB/ECH.1904, 31.I.2011; 1spec., UFPB/ECH.1913, 04.IX.2010; Mangrove on highway to Galinhos, 1spec., UFPB/ECH.1914, 22.VI.1982. Paraíba: 7°03'50"S; 34°47'19"W, 2spec., UFPB/ECH.568, 21.III.2006; Lucena, Fagundes Beach, 2spec., UFPB/ECH.717, 22.IX.1995, 1spec., UFPB/ECH.728, 22.IX.1985; Cabedelo, Areia Vermelha reef, 2spec., UFPB/ECH.1464, 06.IV.2008; Cabedelo, Poço Beach, reefs facing Ponta de Campina, 1spec., UFPB/ECH.1903, 28.II.2010; João Pessoa, 7°4'30” S; 34°46'56”, 1spec., UFPB/ECH.725, 26.IV.2005; João Pessoa, Cabo Branco Beach, 3spec., UFPB/ECH.704, 2002, 4spec., UFPB/ECH.705, 9spec., UFPB/ECH.706, 04.IV.1981, 4spec., UFPB/ECH.707, 03.VII.1985, 1spec., UFPB/ECH.708, 17.II.1980, 1spec., UFPB/ECH.710, 01.IV.2006, 1spec., UFPB/ECH.711, V.1980, 2spec., UFPB/ECH.713, 06.XI.1983, 10spec., UFPB/ECH.714, 29.IV.2002, 3spec., UFPB/ECH.715, 13.XII.1985, 1spec., UFPB/ECH.719, 22.IX.1980, 3spec., UFPB/ECH.722, 09.II.2001, 1spec., UFPB/ECH.727, 09.IX.2006, 3spec., UFPB/ECH.870, 25.XI.2007, 1spec., UFPB/ECH.1240, 22.IX.1980, 2spec., UFPB/ECH.1466, 13.XI.2008, 1spec., UFPB/ECH.1911, 21.III.2000; João Pessoa, Ponta Seixas, 6spec., UFPB/ECH.139, 03.XI.1982, 3spec., UFPB/ECH.703, 23.X.1984, 2spec., UFPB/ECH.712, 25.V.1998; Seixas reef, 1spec., UFPB/ECH.1172, 23.III.2008, 6spec., UFPB/ECH.1234, 03.XI.1982. 4spec., UFPB/ECH.1184, 22.XII.2007, 2spec., UFPB/ECH.1463, 12.I.2009. Pernambuco: Goiana, Catuama Beach, 1spec., UFPB/ECH.1912, 31.X.1982. UFPB/ECH.873; Tamandaré, Carneiros Beach, 4spec., 15.X.1981. Alagoas: Marechal Deodoro, Francês Beach, 1spec., UFPB/ECH.721, 29.I.1983. Bahia: Itaparica, Pedrão de Itaparica, 8spec., UFPB/ECH.716, 18.IX.1982; Itaparica, Ponta de Aratuba, 7spec., UFPB/ECH.723, 19.X.1982; Itaparica, Barra Grande, 2spec., UFPB/ECH.726, 19.IX.1982; Prado, coral reef between Camaruxatiba and Ponta de Imbaçuaba, 3spec., UFPB/ECH.709, 14.X.1982, 2 spec., UFPB/ECH.724, 28.XI.1982; Santa Cruz da Cabrália, Ponta da Coroa Vermelha, 5spec., UFPB/ECH.720, 15.X.1982.

###### Type-locality.

‘Amérique du Nord’ (lectotype) (Clark and Downey 1992).

###### Description.

Disk small (Fig. 11f). Body convex dorsally and flattened ventrally. Five (rarely three to six) short and thick arms, with a robust skeleton (Fig. 11f, g). Abactinal figs mammiform (Fig. 11j), with one large spine. Seven to 11 series of dorsal longitudinal spines. Anus normally positioned within central pentagone of disk and surrounded by 4–6 spines. Madreporite circular with numerous small granules and irregular grooves. Papular areas more numerous on abactinal and lateral regions of arms (Fig. 11h). Papular areas on actinal surface small and widely dispersed. Adambulacral figs with 3 to 4 spines arranged transversally (Fig. 11i).

**Colour.** Red, orange-red, becoming brownish-red when conserved in alcohol and dark brown when dry (Verril 1915, Hendler et al. 1995).

###### Distribution.

Florida, the Bahamas, Gulf of Mexico, Puerto Rico, Jamaica, Nicaragua, Colombia, Venezuela, and Brazil (Alvarado 2011, Benavides-Serrato et al. 2011). In Brazil: CE, RN, PB, PE, AL, BA, ES, and RJ, including Abrolhos (Verrill 1915, Krau 1950, Brito 1962, Lima-Verde 1969, Tommasi 1970, Avila-Pirez 1983, Fernandes et al. 2002, Magalhães et al. 2005, Miranda et al. 2012). From 0 to 55 m in depth (Hendler et al. 1995).

###### Remarks.

Echinaster (Othilia) echinophorus differs from Echinaster (Othilia) sentus for having few large and conspicuous spines on arms and a uniform colour (Hendler et al. 1995). It differs from Echinaster (Othilia) brasiliensis for having thicker arms and mammiform abactinal figs. According to Atwood (1973), Echinaster (Othilia) echinophorus appears to contain several morphologically distinct forms or closely related species. For Walenkamp (1979) the number of series of dorsal longitudinal spines, which Perrier considered to be the main character for separating species in the genus, is quite variable. The examined specimens have a broad morphological variation, among which: number of rows of dorsal longitudinal spines (from 7 to 9), number of spines surrounding the anus (from 4 to 6) and number of granules on the madreporite (from 8 to 15). Notwithstanding, these morphological variations do not seem to be related to ontogenetic development, because even though specimens examined included both juvenile and adult individuals, the differences also affected specimens of the same size. An excellent discussion of the synonymies proposed for the different species of Echinaster (Othilia) echinophorus and of the taxonomic history of the species is found in Clark and Downey (1992) and Walenkamp (1979).

###### Ecological notes.

Lives usually in environments containing consolidated substrates (Hendler et al. 1995), and may be found in estuarine regions (Nobre and Campos-Creasey 2000). It feeds preferably on incrustating organisms belonging to the epifauna and on organic detritus deposited in the substrate (Jangoux and Lawrence 1982). Kempf (1966) found that Echinaster (Othilia) echinophorus may occur in salinities up to 47. We found it in salinities of 52 (Tubarão River/Rio Grande do Norte), forming dense populations. Alves and Dias (2010) commented on the use of this species for medicinal purposes and Machado et al. (2008) remarked that one of the main threats relates to its collecting for aquarists. Presently it is included among the Brazilian species vulnerable to extinction (Machado et al. 2008).

### Order Forcipulatida Perrier, 1884

#### Family Asteriidae Gray, 1840

##### Coscinasterias tenuispina

Taxon classificationAnimaliaForcipuladitaAsteriidae

(Lamarck, 1816)

Figure 9i–m

Asterias tenuispina Lamarck, 1816: 561–562.
Asteracanthion tenuispinus Müller & Troschel, 1842: 16.
Asterias atlantica Verrill, 1868: 368. Rathbun 1879: 145.
Asterias (Stolasterias) tenuispina Sladen, 1889: 565, 583.
Polyasterias tenuispina Perrier, 1894: 108.
Lytaster inaequalis Perrier, 1894: 98–99.
Coscinasterias tenuispina Verrill, 1914: 45. Brito 1960: 4; 1962: 2. Netto 2006: 34, fig. 16c, pl. 5b. Ventura et al. 2007: 228.
Coscinasterias tenuispina var. atlantica Verrill, 1915: 20–21. Tommasi 1966: 24–244.
Stolasterias tenuispina Verrill, 1907: 324.
Coscinasterias (Stolasterias) tenuispina Fisher, 1926: 197.

###### Material examined.

Rio de Janeiro: Cabo Frio, Formoso Beach, 1 spec., MZUSP (without registration number), VII.1956; Cabo Frio, Arraial do Cabo, Brava Beach, 1spec., MZUSP (without registration number), 29.I.2001.

###### Type locality.

‘I’ ocean eropéen’ (Clark and Downey 1992).

###### Description.

Disk small, with 1–3 madreporites (in some cases up to 5 were observed). Six to nine (rarely 5 and unusually 7) thin, elongate (Fig. 9i, j), cylindrical arms, usually of different sizes, the larger ones grouped to one side and the smaller ones to the opposing side. Abactinal figs with one long, conical and pointed spine (~2.17 mm), with base densely surrounded by bivalve pedicallariae with overlapping valves (Fig. 9l). Carinal figs arranged in a regular series. Dorso-lateral figs forming a reticulum. Papular regions occuring on the abactinal and actinal surfaces. Among the abactinal figs, mainly in the intermediate areas, large bivalve pedicellariae are found. Pedicellariae of dorsal spines sessil and with overlapping bivalves, with denteate margins. Oral pedicallariae bivalve (Fig. 9m), consisting of a basal piece into which two valves with smooth margins fit in.

**Colour.** Specimens from Brazil vary from brown to orange colour (Ventura et al. 2007). According to Clark and Downey (1992), specimens from the Mediterranean are usually yellow or whitish-yellow with black or brown spots on abactinal surface and crowns of rusty-red pedicellariae. Individuals from the Bermudas, on the other hand, with dorsal surface purple and the oral surface yellow, blue or violet (Verrill 1915).

###### Distribution.

North Carolina, Gulf of Mexico, Antilles, Bermudas, Cuba, Brazil, Portugal, Spain, France and Montenegro (Tommasi 1970, Downey 1973, Clark and Downey 1992, Alves et al. 2002, Waters and Roy 2003, Kascelan and Mandic 2007). In Brazil: BA, ES, and SP, including Abrolhos (Rathbun 1879, Verrill 1915, Brito 1960, Tommasi 1970, Ventura et al. 2007). Intertidal to 165 m in depth (Clark and Downey 1992).

###### Remarks.

Two species of the genus *Coscinasterias* Verrill, 1870 are known for the Atlantic, *Coscinasterias tenuispina* and *Coscinasterias calamaria* (Gray, 1840). The first is widely distributed through the Atlantic and Mediterranean, while the second is restricted to South Africa, Angola, and Madagascar (Mah 2013). *Coscinasterias tenuispina* differs from *Coscinasterias calamaria* for having intercrossing pedicellariae with a well developped terminal tooth (except in some specimens from Brazil). Clark and Downey (1992) suggested a subspecific distinction for these species on the basis of morphological similarities observed in Brazilian and South African specimens. However, according to Waters and Roy (2003) these observations were made on the basis of juvenile specimens and it is thus necessary to undertake new morphological analyses to clarify the close relationships between *Coscinasterias calamaria* and *Coscinasterias tenuispina* (Waters and Roy 2003). In the phylogeographic analysis of Waters and Roy (op. cit.) for species of *Coscinasterias*, morphological variations were observed between populations from Brazil on the one side and from the Bermudas and the Mediterranean on the other. No morphological variations are observed in the specimens examined by us.

###### Ecological notes.

Lives in consolidated substrates, including areas with strong hydrodynamism (Machado et al. 2008). *Coscinasterias tenuispina* has extra-oral digestion and feeds on epifaunal organisms, mainly mussels (Ventura et al. 2007). It is a fissiparous species, which presents an annual gonadal cycle and a long period of spawning (Alves et al. 2002). According to these authors, the preponderance of males in the population of Itaipu Beach (Niterói/Rio de Janeiro) suggests that assexual reproduction by fission is predominant and, consequently, that the number of clones must be significant. According to Brito (1962), this species is very common in Cabo Frio (Rio de Janeiro). However, since the first record by Rathbun (1879) of *Coscinasterias tenuispina* for Abrolhos (Bahia), the species has not been cited again off the northeast region of Brazil. As pointed out by Machado et al. (2008), although this species has a wide geographical distribution, its range is discontinuous, probably due to its assexual reproduction, that limits dispersal ability. Presently the species is listed among those vulnerable to extinction, having among the main causes of population decline the constant destruction of its habitat, the erosion of the substrate, the effects of pollutants, the precarious sanitation and the excess of tourists and divers within their range of occurrence (Machado et al. 2008).

## Discussion

The fauna of Asteroidea recorded for northeastern Brazil is composed mainly by species with broad geographical and bathimetic distributions, and considered common species for the Brazilian littoral (Tommasi 1970). One exception is *Mithrodia clavigera*, which represents a new record for the northeast and is typically a species of deeper waters. Another two species represent new records for northeast Brazil: *Astropecten alligator* and *Luidia ludwigi scotti*.

Among the four recorded orders, Paxillosida was the nost diverse (n = 10 spp), followed by Valvatida (n = 5 spp), Velatida (n = 3 spp), Spinulosida (n = 2 spp) and Forcipulatida (n = 1 spp). These results were expected, because Paxillosida represents the most diverse order and contains the most speciose genus (*Astropecten* with 150 spp) and abundant species in shallow waters (Zulliger et al. 2010). Although common in marine communities, the taxonomy of the species composing this order and the phylogenetic relationships of the Paxillosida are still uncertain and contradictory (Matsubara et al. 2005). During many years this order was considered to be the most primitive in the class, due mainly to the absence of an anus and of ventosae on the ambulacral feet (Jangoux 1982). However, a reexamination of characters evidenced that these characters represent adaptations to life in sandy environments that produced character losses instead of being primitive absences (Matsubara et al. 2005).

The genus *Astropecten* represents one of the most complex taxa within the class Asteroidea, in which species exibit great morphological plasticity, making identification of species difficult. According to Zulliger et al. (2010), the high phenotypic variability of this genus resulted in the naming of several subspecies. Six of these were recognized in Brazil (Tommasi 1970). Presently they are all synonymized (Clark and Downey 1992; Mah 2013) and the records of *Astropecten armatus* for the Brazilian coast represent synonyms of *Astropecten brasiliensis* (Tommasi 1999, unpublished data). Among the several taxonomic characters used for the identification of species, the appearance of the paxillae and of the superomarginal figs, together with the number and shape of the spines of the marginal fringe are the characters that contribute most for the identification of species. On the other hand, the number and shape of the adambulacral spines, characters that were much used by authros such as Bernasconi (1955, 1957), Tommasi (1970) and Clark and Downey (1992), proved to be very similar among specimens and were thus not considered to be good characters for the taxonomy of the genus.

Another taxonomicly complex genus is *Echinaster* that, similarly to *Astropecten*, presents large morphological variability, making species identification difficult. According to Clark and Downey (1992), the species of this genus occurring in Brazil are polymorphic, with possible hybridization among them. Presently three occurrences of Echinaster (Othilia) are considered valid along the Brazilian coast: Echinaster (Othilia) brasiliensis, Echinaster (Othilia) echinophorus, and Echinaster (Othilia) guyanensis. According to Avila-Pires (1983), the first occurs only in the south and southeastern regions, below Espírito Santo, and the second is restricted to the northeastern region. That author does not confirm the presence of Echinaster (Othilia) guyanensis, but Clark and Downey (1992) indicate its distribution as being from Central America to Belém (Pará). Magalhães et al. (2005) recorded Echinaster (Othilia) guyanensis for the littoral of Bahia, but this occorrence could not be confirmed in the present study. On the basis of the material we examined we cannot agree with Avila-Pires (1983) that Echinaster (Othilia) brasiliensis does not occur in the northeastern region. We confirm its presence in northeastern Brazil, although it is rarer there than its congener Echinaster (Othilia) echinophorus. Among the several characters used in the taxonomy of the genus, the aspect of the endoskeleton figs represent one of the most important criteria for species recognition. The number of spine rows on the arms was used by Avila-Pires (1983) and Bernasconi (1957) to separate species, but it turned out be be quite variable in this study.

In terms of diversity, the fauna of asteroids in northeastern Brazil represent only 27% of the species known for Brazil, the coast of Bahia (n = 14 spp) and Paraíba (n = 12 spp) being the most diverse, followed by Pernambuco (n = 9 spp), Ceará and Rio Grande do Norte (both with 6 spp), Alagoas (n = 4 spp), and Piauí (n = 2 spp). There are still no records of asteroids for the coasts of Maranhão and Sergipe. These numbers indicate the scarce knowledge available on the Asteroidea from northeast Brazil, which represents one of the least known areas regarding the benthic macrofauna (Ventura et al. 2007). The species refered for northeast Brazil (n = 21 spp) are mostly shallow water species (up to 30 meters in depth), with only 4 species representing deep-water forms. It remains crucial to conduct inventories in little explored areas such as northeast Brazil, particularly in deep waters.

Presently 374 species of sea-stars are known for the Atlantic Ocean (Clark and Downey 1992), the Gulf of Mexico being the most speciose area, with 126 recorded species (Pawson et al. 2009), followed by the Caribbean, with 116 spp (Alvarado et al. 2011). The fauna from Brazil represents only 20.5% of the known species from the Western Atlantic, while those from northeast Brazil represent 4.82%. Considering the similarity of the Brazilian fauna with the Caribbean region, and taking into account the extent of the Brazilian coastline, the necessity to make an inventory and to describe the Brazilian fauna becomes urgent. According to Marques and Lamas (2006), the degree of knowledge of the marine fauna from Brazil is far from ideal and the most notable gap in our knowledge refers to the the invertebrates mainly from northeast Brazil.

Another three species recorded previously for northeast Brazil were not confirmed in the present study: *Allostichaster hartti* (as *Leptasterias hartii* Rathbun, 1879) was recorded as a rare species for the littoral of the State of Bahia (Brito 1962); *Asterina stellifera* (Möbius, 1859) (as *Enoplopatiria marginata* (Hupe, 1857)) was cited by Bernasconi (1955) for the Abrolhos Archipelago and Echinaster (Othilia) guyanensis was recorded by Magalhães et al. (2005) for the coast of Bahia. Ventura et al. (2007) cited *Astropecten acutiradiatus*, *Nymphaster arenatus* and *Plinthaster dentatus* for the continental shelf of Bahia. Unfortunately, we have been unable to retrieve these species in the present study.

Due to the negligible previous knowledge on the Asteroidea from the littoral of northeastern Brazil, the present study represents an important re-evaluation of the diversity of this group for this area. It should further be noted that the study of material deposited in scientific collections turned out to be of fundamental importance, permitting a historical panorama of the Asteroidea from northeast Brazil. Deep-water sampling in the studied region, restricted to sporadic expeditions, as accounted for in Clark and Downey (1992), has still not provided many published records on asteroids. The diversity of the deep-water and abyssal marine fauna of the South West Atlantic perhaps remains the least known in the world, and clearly represents the next marine frontier to be systematically sampled and studied, both for asteroids and for other marine groups.

## Supplementary Material

XML Treatment for Luidia alternata alternata

XML Treatment for Luidia clathrata

XML Treatment for Luidia ludwigi scotti

XML Treatment for Luidia senegalensis

XML Treatment for Astropecten alligator

XML Treatment for Astropecten articulatus

XML Treatment for Astropecten brasiliensis

XML Treatment for Astropecten cingulatus

XML Treatment for Astropecten marginatus

XML Treatment for Asterinides folium

XML Treatment for Mithrodia clavigera

XML Treatment for Oreaster reticulatus

XML Treatment for Calyptraster coa

XML Treatment for Linckia guildingi

XML Treatment for Narcissia trigonaria

XML Treatment for Echinaster (Othilia) brasiliensis

XML Treatment for Echinaster (Othilia) echinophorus

XML Treatment for Coscinasterias tenuispina
